# Isopentenol Utilization Pathway for the Production of Linalool in *Escherichia coli* Using an Improved Bacterial Linalool/Nerolidol Synthase

**DOI:** 10.1002/cbic.202100110

**Published:** 2021-05-25

**Authors:** Clara A. Ferraz, Nicole G. H. Leferink, Iaroslav Kosov, Nigel S. Scrutton

**Affiliations:** ^1^ Manchester Institute of Biotechnology, Department of Chemistry School of Natural Sciences University of Manchester 131 Princess Street Manchester M1 7DN UK; ^2^ Future Biomanufacturing Research Hub Manchester Institute of Biotechnology, Department of Chemistry School of Natural Sciences University of Manchester 131 Princess Street Manchester M1 7DN UK

**Keywords:** isopentenol utilization pathway, linalool, protein engineering, synthetic biology, terpenoids

## Abstract

Linalool is a monoterpenoid used as a fragrance ingredient, and is a promising source for alternative fuels. Synthetic biology offers attractive alternative production methods compared to extraction from natural sources and chemical synthesis. Linalool/nerolidol synthase (bLinS) from *Streptomyces clavuligerus* is a bifunctional enzyme, producing linalool as well as the sesquiterpenoid nerolidol when expressed in engineered *Escherichia coli* harbouring a precursor terpenoid pathway such as the mevalonate (MVA) pathway. Here we identified two residues important for substrate selection by bLinS, L72 and V214, where the introduction of bulkier residues results in variants with reduced nerolidol formation. Terpenoid production using canonical precursor pathways is usually limited by numerous and highly regulated enzymatic steps. Here we compared the canonical MVA pathway to the non‐canonical isopentenol utilization (IU) pathway to produce linalool using the optimised bLinS variant. The IU pathway uses isoprenol and prenol to produce linalool in only five steps. Adjusting substrate, plasmid system, inducer concentration, and cell strain directs the flux towards monoterpenoids. Our integrated approach, combining enzyme engineering with flux control using the artificial IU pathway, resulted in high purity production of the commercially attractive monoterpenoid linalool, and will guide future efforts towards efficient optimisation of terpenoid production in engineered microbes.

## Graphical abstract

**Linalool is a monoterpenoid** used as fragrance ingredient, and a promising source for alternative fuels. Here we explore the artificial isoprenol utilization pathway to produce linalool in *Escherichia coli* using the linalool/nerolidol synthase from *Streptomyces clavuligerus*. The generation of improved bLinS variants together with substrate and strain optimization increased linalool production and decreased nerolidol by‐product levels.

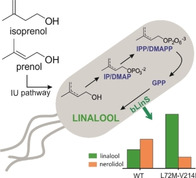

## Introduction

Terpenoids (or isoprenoids) are an abundant and diverse class of natural products with more than 80,000 compounds described in the Dictionary of Natural Compounds.^[1]^ Most terpenoids are commonly found in plants where they exhibit a multitude of biological roles, ranging from species‐to‐species communication, to intracellular signalling, and defence against predatory species.^[2]^ Due to their structural diversity, terpenoids also have a wide range of industrial applications and are used as pharmaceuticals, herbicides, flavourings, fragrances and as alternatives for fossil fuels.^[3]^ Linalool is an odoriferous acyclic terpenoid, predominantly occurring in nature as (*R*)‐(−)‐linalool and contributes to the floral scent in over 200 plant species of different families.^[4]^ In 2019 the global linalool market valued 9,980 million USD, and is expected to grow 3.6 % over the next five years, reaching 12,300 million dollars in 2024.^[5]^ Linalool is widely used in cosmetic products like perfumes, lotions, soaps, and shampoos, as well as in non‐cosmetic household products such as detergents, and cleaning agents. Furthermore, linalool is vital to the manufacturing of Vitamin E.^[6]^ More recently, linalool and other terpenoids have attracted attention as candidates for jet fuel replacements due their low freezing point and high energy density.^[7]^ Limitations, such as low levels of terpenoids produced by plants and naturally occurring microorganisms, stereo‐chemical complexities and use of hazardous solvents for their chemical synthesis, have directed research efforts towards the development of engineered microbes for the production of terpenoids.^[8]^

All terpenoids are naturally synthesized from the C5 building blocks isopentenyl diphosphate (IPP) and dimethylallyl diphosphate (DMAPP). The combination of IPP and DMAPP can generate diphosphate substrates of varying carbon lengths, which can then be utilized by terpenoid synthases to produce monoterpenoids (C10), sesquiterpenoids (C15), diterpenoids (C20), or larger terpenoids. For example, geranyl diphosphate (GPP), the universal substrate for monoterpenoids is synthesized by head‐to‐tail coupling of one molecule each of DMAPP and IPP. The addition of another IPP unit to GPP results in farnesyl diphosphate (FPP), the precursor for all sesquiterpenoids. Eukaryotes and archaea produce IPP and DMAPP through the mevalonate (MVA) pathway, while most bacteria, some eukaryotic parasites and plant chloroplasts use the 2‐*C*‐methyl‐D‐erythritol 4‐phosphate (MEP) pathway. The MVA and the MEP pathways are composed of 18 enzymatic steps to produce IPP or DMAPP from glucose,^[9]^ and both pathways were previously engineered in *Escherichia coli* and other prokaryotic and eukaryotic organisms to produce terpenoids.

Linalool has been successfully produced in engineered yeast or *E. coli* as a host using a heterologous MVA pathway or the endogenous MEP pathway with linalool synthases from plant or bacterial sources.^[10]^ However, plant linalool synthases, when expressed in either yeast or *E. coli*, result in very low product titres (<1 mg L^−1^).[^10a^, ^10b^, ^10c^, ^10d^] The use of a bacterial linalool/nerolidol synthase (bLinS) from *Streptomyces clavuligerus* resulted in much higher linalool titres (up to 500 mg L^−1^).[^10e^, ^10f^] But, because bLinS also accepts FPP as substrate, which is naturally produced by *E. coli*, the sesquiterpene *trans*‐nerolidol is produced as by‐product (∼30 % of total product mixture).^[10f]^ For commercial production the generation of single, clean products is desirable, as this would require less downstream processing.

**Figure 1:**
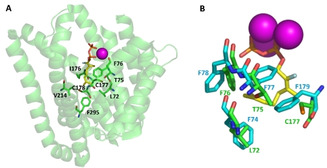
Identification of bLinS target residues from structural analysis of bLinS and bCinS. A) Cartoon representation of the bLinS structure in complex with a fluorinated GPP analogue (PDB: 5NX5^[17]^). Residues identified for mutagenesis are indicated, the fluorinated GPP analogue is shown in yellow sticks and magnesium ions are represented as purple spheres. B) Active site overlay of bLinS (green) and bCinS (PDB: 5NX7,^[17]^ cyan). The fluorinated substrate analogue is shown in yellow sticks and magnesium ions are represented as purple spheres.

One approach to reduce the formation of the nerolidol by‐product is to engineer bLinS so that it no longer accepts FPP as substrate. Structural analysis of bLinS and the related enzyme cineole synthase (bCinS) provides a rationale for the fact that bLinS can accept both GPP and FPP as substrate, while bCinS can only convert GPP. Crucially, the bCinS active site is rich in bulky aromatic residues where bLinS contains smaller, non‐aromatic, residues resembling bacterial sesquiterpene synthases. These residues offer attractive protein engineering targets aiming at active site restriction for improved linalool production (Figure 1,^[10f]^).

Alternatively, the metabolic flux can be directed towards monoterpenoids over larger terpenoids. The *E. coli* FPP synthase (IspA) gene is not essential for viability, but FPP is a precursor to essential compounds such as dolichols and respiratory quinones,^[11]^ and as such, IspA knock‐out mutants show a substantial reduction in growth rate.^[11]^ Removal of FPP from the substrate pool via an IspA knock‐out in the *E. coli* terpenoid production strain is therefore not a viable strategy to prevent nerolidol formation by bLinS. However, using alternative non‐canonical isoprenoid precursor pathways the flux may be directed away from FPP towards GPP.

**Scheme 1:**
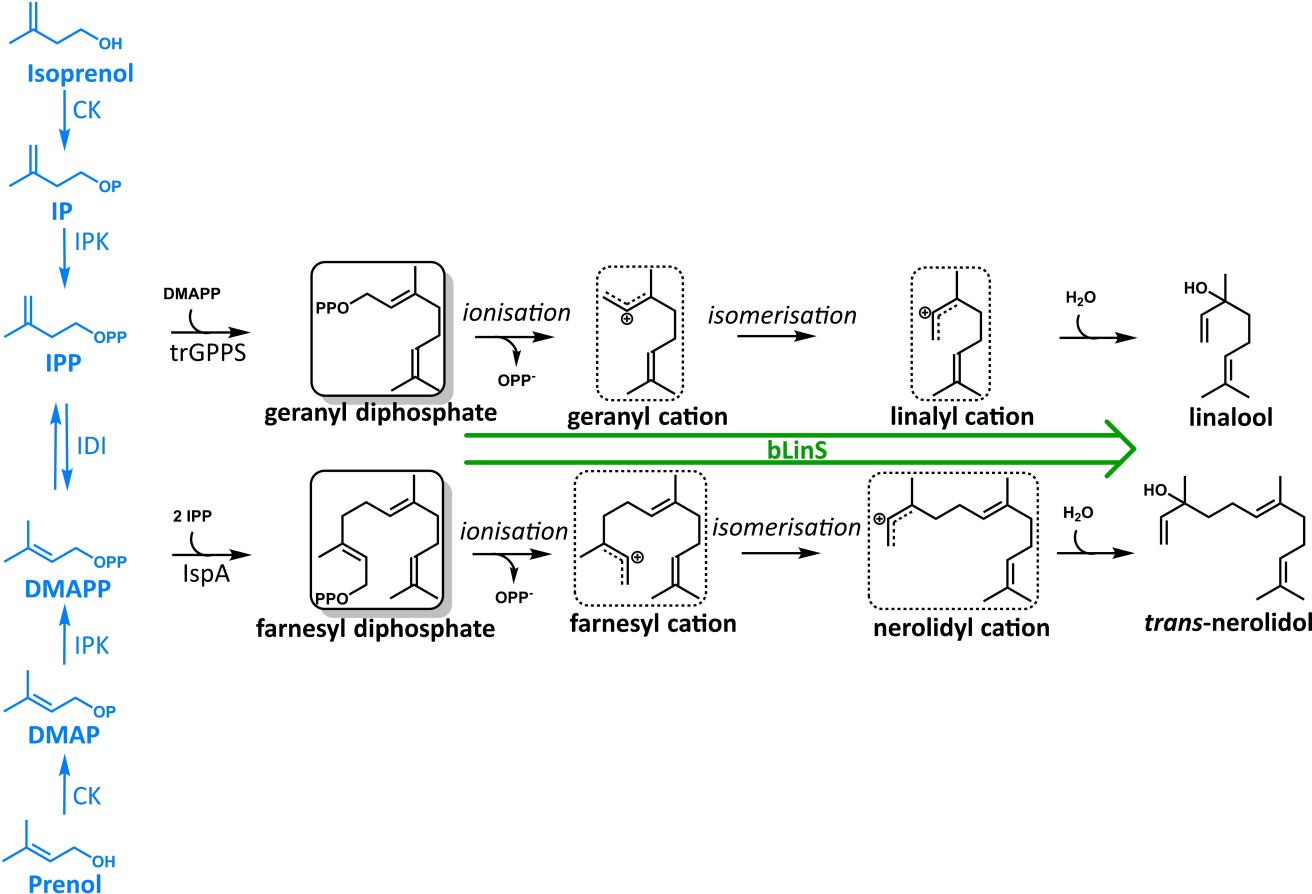
IU pathway and the proposed mechanism for the formation of linalool and *trans*‐nerolidol in the engineered *E. coli* strain using the GPPS‐bLinS module. Isoprenol and prenol are phosphorylated by choline kinase (CK) producing isopentenyl phosphate (IP) and dimethylallyl phosphate (DMAP), respectively. A second phosphorylation is catalysed by isopentenyl phosphate kinase (IPK), producing isopentenyl diphosphate (IPP) and dimethylallyl diphosphate (DMAPP), which are isomerized by isopentenyl diphosphate delta‐isomerase (IDI), shown in blue. The C5 isoprenoid precursors are converted to the mono‐ and sesquiterpenoid substrates geranyl diphosphate (GPP) and farnesyl diphosphate (FPP) by a heterologous truncated GPP synthase (trGPPS) and an endogenous FPP synthase (IspA) respectively. bLinS accepts both GPP and FPP which are converted to linalool and *trans*‐nerolidol, respectively. Carbocation reaction intermediates are highlighted with dashed boxes. The isomerisation steps occur via linalyl diphosphate and nerolidyl diphosphate.

Pathways orthogonal to the central metabolism can be more effective for chemical production as they can bypass highly regulated steps, avoid toxic intermediate accumulation and reduce carbon loss related to precursor synthesis.^[12]^ Alternative non‐canonical pathways that bypass the MVA pathway were recently developed for this purpose.^[13]^ One strategy consists of using isoprenol (3‐methyl‐3‐buten‐1‐ol) or prenol (3‐methyl‐2‐buten‐1‐ol) alcohols as feedstock to produce IPP and DMAPP in a pathway composed of only two or three enzymes. This pathway was named by different groups as isopentenol utilization (IU) pathway,^[14]^ alcohol‐dependent hemiterpenoid pathway,^[15]^ isoprenoid alcohol pathway^[13]^ or terpenoid mini‐path,^[16]^ depending on the enzymes used. The IU pathway uses *Saccharomyces cerevisiae* choline kinase promiscuous activity to phosphorylate isoprenol and prenol, *Arabidopsis thaliana* isopentenyl phosphate kinase for the second phosphorylation step and *E. coli* isopentenyl‐diphosphate delta‐isomerase to interconvert IPP and DMAPP (Scheme 1). By supplying different amounts of isoprenol and prenol, it is possible to direct the biosynthesis towards the terpenoid of interest, as IPP and DMAPP are consumed at different ratios depending on the product of interest.^[9]^

Here we compared linalool production using the canonical MVA pathway to the artificial IU pathway using an integrated approach, combining protein engineering with flux control to improve titres and product purity in *E. coli*. We identified two important residues (L72 and V214) for GPP or FPP substrate selection by bLinS. Their exchange for bulkier residues resulted in bLinS variants with reduced nerolidol production. The IU pathway uses isoprenol and prenol to produce linalool in only five steps, and adjusting substrate concentration directs the flux towards monoterpenoids. Plasmid, inducer and strain optimisations further increased linalool titres. The optimised IU pathway produced 167 mg L_org_^−1^ linalool with only 17 % nerolidol in the total product mixture.

## Results and Discussion

### bLinS engineering

Previously we speculated that because bLinS contains smaller, non‐aromatic, residues at positions equivalent to bCinS Phe77 and Phe179 (Thr75 and Cys177 in bLinS), it resembles the sesquiterpenoid synthases aristocholene synthase and selinadiene synthase. This could explain why bLinS accepts GPP and FPP as substrates whilst bCinS, with a smaller active site, only converts GPP.^[17]^ Structural analysis of bLinS and the related bCinS (Figure 1), revealed several candidate amino acid residue positions that could restrict access of FPP to the active site of bCinS but not bLinS. The product profile of native bLinS expressed in the engineered *E. coli* strain was determined previously^[17]^ by GC‐MS analysis of the organic overlay (see Figure S1 in the Supporting Information). The obtained linalool titre was 360 mg L_org_^−1^, which constitutes approximately 65 % of all terpenoids collected in the organic layer, nerolidol was 29 % of the total and geraniol and derivatives, produced as a result of endogenous *E. coli* activity together constituted about 6 % of the total terpenoid production.

Residues Leu72, Thr75 and Cys177 were selected because the equivalent positions in the related bCinS enzyme contain a bulky phenylalanine residue (Figure 1B). In addition, residues Leu72 and Thr75 align to a previously identified plasticity region that is partly responsible for product outcome in plant monoterpene cyclases/synthases.^[18]^ Residues Ile176, Cys178, Val214 and Phe295 were selected because of their position at the bottom of the active site, and side‐chain orientation towards the substrate analogue in the crystal structure, which potentially allows for a reduction in the size of the active site cleft via the introduction of larger amino acids, thereby preventing the binding of FPP via steric hindrance (Figure 1A).

All bLinS variants created were introduced in a pGPPS‐bLinS plasmid comprising *Abies grandis* GPPS and bLinS, in an *E. coli* strain harbouring the pMVA plasmid^[10a]^ for heterologous expression of the MVA pathway. This allows the rapid determination of full product profiles without the need for expensive GPP and FPP substrates and laborious protein purification steps.^[19]^ Variant strains were grown in two‐phase shake‐flask cultures using glucose as the carbon source and *n*‐nonane as the organic phase to trap the volatile terpenoids produced. In the first round of mutagenesis a bulky phenylalanine (Phe, F) residue was introduced at one or more positions to replace Leu72, Thr75, and Cys177 to mimic the smaller active site cavity of bCinS. Full product profiles of the variants were obtained upon insertion of the variants in our previously established MVA‐dependent monoterpenoid production platform^[19]^ (see Figures S6, S11 and Table S6 in the Supporting Information). Geraniol and farnesol are not produced by purified native bLinS *in vitro*,^[17]^ but by endogenous *E. coli* activity, and are thus ‘reporter’ products for the availability of both GPP and FPP in the substrate pool. Interestingly most *E. coli* strains harbouring these variants did not produce linalool or nerolidol. Only the strain containing variant L72F produces a very small amount of linalool (<3 mg L_org_^−1^) all other strains only produce geraniol and derivatives at concentrations ranging from 10–35 mg L_org_^−1^, which is the result of endogenous *E. coli* activity.[^18^, ^20^] Introduction of Phe at positions Ile176, Cys178 and Val214, and a tryptophan (Trp, W) at position Phe295 had a similar effect, and expression in the monoterpenoid production platform resulted in strains that only produced geraniol and derivatives, suggesting that neither FPP nor GPP are accepted as substrates in these bLinS variants.

A second round of mutagenesis was conducted at the same amino acid positions, introducing amino acids that are bulkier than the original residues. A methionine residue (Met, M) was introduced at positions Leu72, Thr75 and C178, a leucine (Leu, L) residue was introduced at position Val214, and a tyrosine (Tyr, Y) residue was introduced at position Phe295. All *E. coli* strains harbouring these second round bLinS variants were capable of producing linalool, albeit at a very low level for variants T75M, C178M and F295Y (1–5 mg L_org_^−1^) (see Figure S7 and Table S6 in the Supporting Information). Two variants show favourable product profiles; variant L72M has a linalool production level similar to native bLinS (430 mg L_org_^−1^), but a lower nerolidol production (8 % of total terpenoids production). Variant V214L, has a relatively low linalool titre compared to native bLinS (50 mg L_org_^−1^), but the relative nerolidol production is even lower (<2 %).

**Figure 2:**
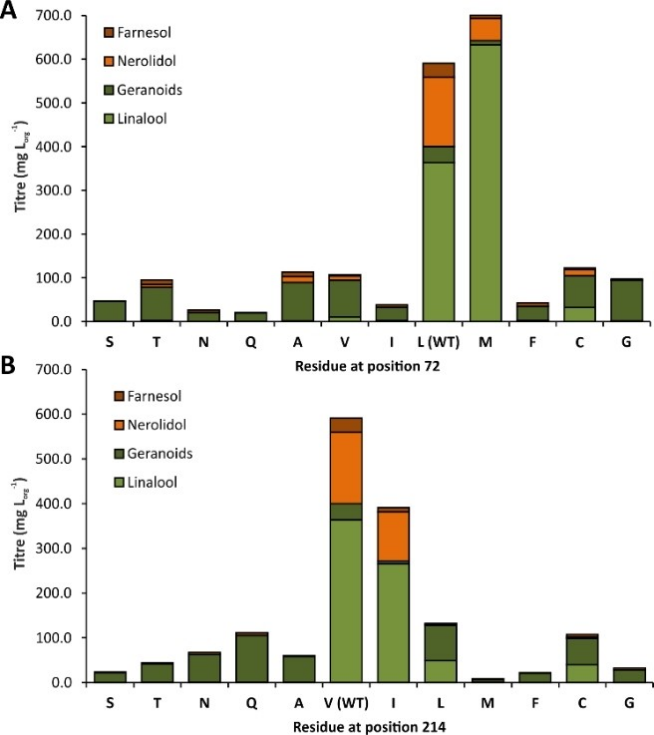
Product profiles and titres obtained for bLinS variants with single mutations at position 72 (A) and 214 (B). Product profiles obtained when expressed in engineered *E. coli* for terpenoids production. Full product profiles, titres, and standard deviations are shown in Table S6 of the Supporting Information. WT is wild‐type bLinS.

To further investigate the role of amino acid composition at positions 72 and 214 in substrate acceptance and product outcome, additional single and double variants were constructed. First, either L72 or V214 were replaced with a variety of amino acids, to investigate if the size and polarity of the residues at these positions are important. Both L72 and V214 can be replaced with a limited number of hydrophobic residues of medium size, resulting in active variants that still produce linalool (see Figure 2 and Figures S8, S9 and Table S6 in the Supporting Information). The best variants, L72M, V214I, and V214L were combined in two double variants, both of which display favourable properties over the native enzyme (Figure S12). Variant L72M produces the highest relative linalool content (>90 % of total terpenoids), with linalool titres that are higher than observed for native bLinS (630 mg L_org_^‐1^ vs 360 mg L_org_^−1^). L72M‐V214I has a similar relative product profile to native bLinS, but expression in our platform results in even higher linalool titres (up to 1 g L_org_^−1^), and L72M‐V214L has the highest linalool/nerolidol ratio, a cumulative effect of both single mutations (see Figure S12 in the Supporting Information). Residues at positions 72 and 214 both need to be hydrophobic for linalool production, and size cannot deviate extensively from the original residue. This suggests the effect of these residues on substrate acceptance is of a steric nature. This is consistent with observed product profiles of variants where a smaller residue is introduced (e. g. L72A, L72T) which produce more nerolidol than linalool. See Table S6 in the Supporting Information for a full breakdown of all product profiles obtained in this study. Interestingly, a recently discovered new clade of fungal linalool/nerolidol synthases contains members that are capable of producing clean linalool product profiles, even in the presence of FPP substrate.^[21]^ Residues involved in substrate specificity in these fungal enzymes map to a different part of the active site, close to the negatively charged diphosphate group of the substrate, whereas the residues identified in this study interact only with the hydrocarbon moiety of the substrate. However future structural work is needed to confirm this.

**Figure 3:**
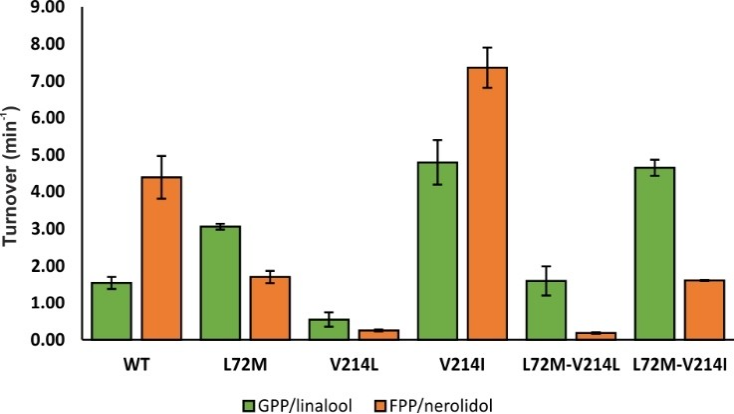
*In vitro* biotransformations of selected bLinS variants with excess GPP and FPP. The average value and SD of two replicates are shown. No geraniol or farnesol was detected in any of the reaction mixtures, confirming that the source of geraniol and farnesol in *in vivo* production experiments is endogenous *E. coli* activity.

The *in vivo* product profiles were confirmed by *in vitro* biotransformations on GPP and FPP with selected purified bLinS variants (Figure 3). Interestingly, even though in the *in vivo* experiments more linalool is produced than nerolidol, it appears that WT bLinS actually prefers FPP over GPP. This suggests that using the heterologous MVA pathway the intracellular GPP concentration is higher than FPP, despite the presence of the endogenous IspA gene. Variants that contain either the L72M or V214L mutation prefer GPP over FPP, confirming their role in substrate selection in bLinS. These studies also confirmed that endogenous *E. coli* activity is the source of any detected geraniol and farnesol, and are not the result of mutations introduced in the bLinS gene.

### IU pathway

Non‐canonical precursor pathways have the potential to increase terpenoid titres by overcoming inherent regulation of the MEP and MVA pathways by increasing orthogonality. Alternative pathways which use isoprenol and prenol as substrates were recently exploited for mono‐, sesqui‐, di‐ and tetraterpenoids production and showed to be a promising strategy for high‐titre terpenoid production in *E. coli*.[^13^, ^14^, ^15^] The IU pathway was compared to the MVA pathway for linalool production using the WT and the best performing bLinS variants.

### Isoprenol and prenol toxicity

**Figure 4:**
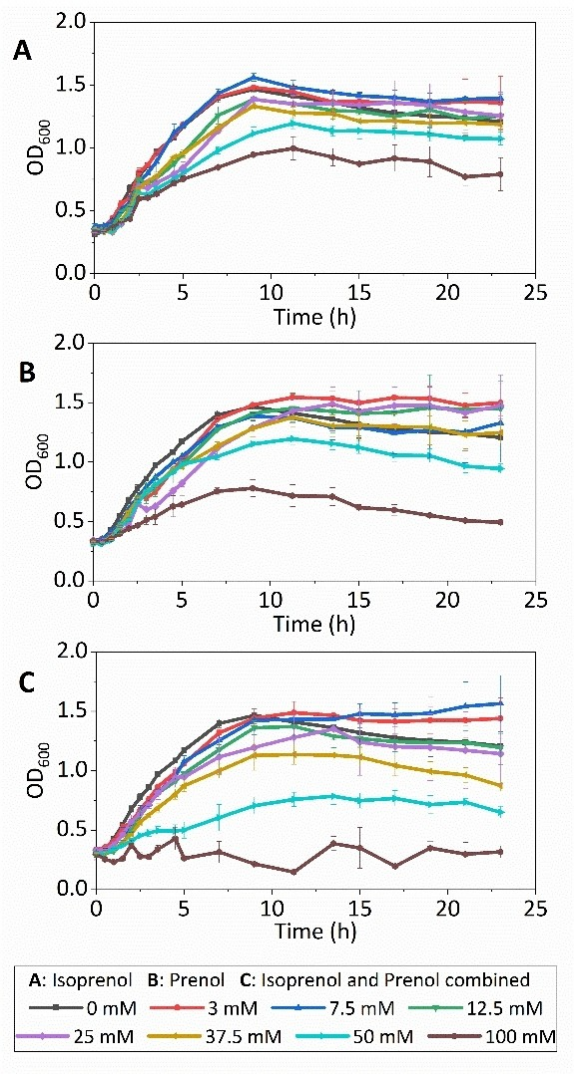
Isoprenol and prenol toxicity in *E. coli* in TB medium under different concentrations. Overnight *E. coli* NEB‐5α cells were sub‐cultured and grown until OD_600_=0.3–0.4. Isoprenol and prenol were added at different concentrations and toxicity was determined through culture growth in a 200 μL 96‐well plate at 30 °C by measuring the OD_600_ for 24 h. Error bars represent the standard deviation of three to four biological replicates.

Previous reports have demonstrated the toxicity of short alcohols to microbial hosts, affecting not only cell growth but also product titre.^[22]^ Before implementing the IU pathway to produce linalool, isoprenol and prenol toxicity in *E. coli* was evaluated. Measurements of optical density at 600 nm (OD_600_) were performed at increasing concentrations of exogenous isoprenol and prenol in terrific broth (TB) medium (Figure 4). Low concentrations of isoprenol or prenol do not affect microbial growth, but the addition of 50 mM and 100 mM of each alcohol individually inhibits cellular growth. A recent study also found the half‐maximal inhibitory concentration of isoprenol to be 53 mM.^[23]^ When the two compounds were added in combination, the growth rate was reduced at 37.5 mM each and the addition of both alcohols at 100 mM each inhibits cell growth almost completely.

### Linalool production using the IU pathway

The genes encoding the IU pathway developed by Chatzivasileiou *et al*.^[14]^ were cloned in the pMVA plasmid, thereby replacing the MVA genes, resulting in the plasmid p(Iso)prenol. This plasmid was co‐transformed into *E. coli* NEB‐5α cells with the plasmid pGPPS‐bLinS. Since a previous study has shown that a single‐plasmid system could perform better than a two‐plasmid system,^[10e]^ a single‐plasmid, p(Iso)prenol‐GPPS‐bLinS was constructed, cloning the genes encoding the IU pathway into the pMVA‐GPPS‐bLinS plasmid as described.

**Figure 5:**
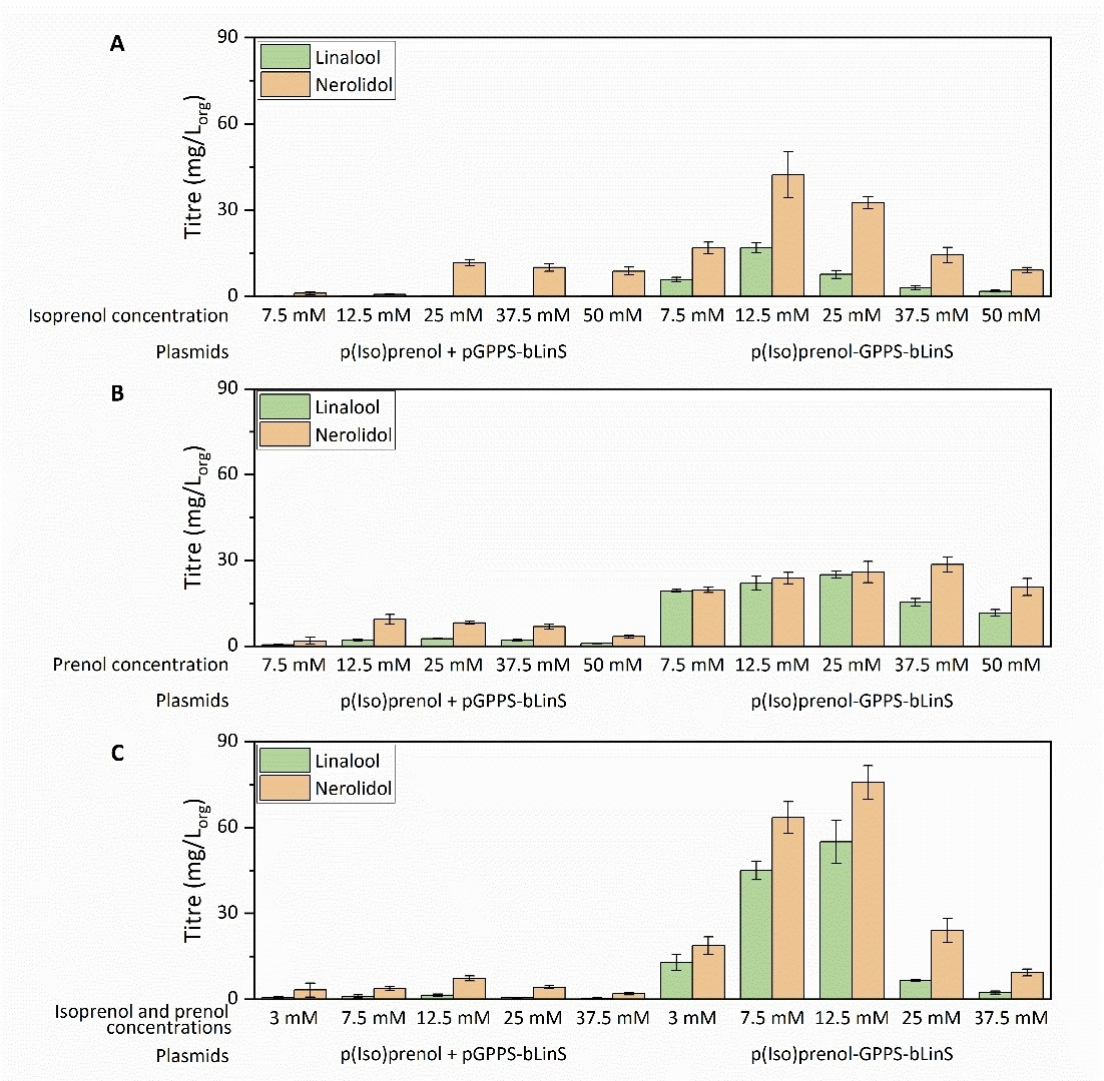
Linalool and nerolidol titres obtained with the IU pathway using a two‐plasmid system (p(Iso)prenol+pGPPS‐bLinS) or a single‐plasmid system (p(Iso)prenol‐GPPS‐bLinS) with different concentrations of isoprenol (A), prenol (B), and isoprenol and prenol together (C). *E. coli* NEB‐5α cells were grown in 3 mL TB media with 20 % *n*‐nonane overlay for 72 h. Error bars represent the standard deviation of three biological replicates.

The two‐ and single‐plasmid systems were tested for linalool production using a concentration of 25 mM isoprenol in the culture medium (concentration optimized by Chatzivasileiou *et al*.^[14]^). Strains were grown in two‐phase shake‐flask cultures using glucose as the carbon source and *n*‐nonane as the organic phase to trap the volatile terpenoids produced. After 72 h, terpenoids were recovered from the organic layer and analysed by GC‐MS. Total terpenoid titres obtained from the IU pathway were substantially lower than the titres obtained with the MVA pathway. Only 0.2 mg L_org_^−1^ linalool was produced using the two‐plasmid system and approximately 8 mg L_org_^−1^ linalool using the single‐plasmid system under the same conditions (Figure 5A). Interestingly, the linalool/nerolidol ratio obtained from the IU pathway was very different from the one obtained using the MVA pathway, although the same terpenoid synthase was used. Nerolidol was produced at higher amounts by both systems, achieving 12 mg L_org_^−1^ in the two‐plasmid system, and 33 mg L_org_^−1^ in the single‐plasmid system (Figure 5A), suggesting a higher availability of FPP over GPP compared to the MVA pathway. This correlates to the *in vitro* results which shows that at an excess of GPP and FPP, WT bLinS prefers the sesquiterpenoid precursor FPP.

Next, isoprenol was supplied at lower and higher concentrations to evaluate the impact on product titres. Although linalool levels did not increase at any condition with the two‐plasmid system, decreasing the isoprenol amount to 12.5 mM doubled the linalool titre to 16 mg L_org_^−1^ in the single‐plasmid system (Figure 5A). In all conditions tested, nerolidol titres were at least doubled compared to linalool, reaching almost 50 mg L_org_^−1^ for the single‐plasmid system supplied with 12.5 mM isoprenol. Total terpenoid titres were reduced at higher substrate concentrations, which is most likely due to increased substrate toxicity.

The nerolidol precursor, FPP, is obtained via the condensation of two units of IPP, the product of isoprenol phosphorylation, and one unit of DMAPP, the product of prenol phosphorylation (Scheme 1). The low linalool/nerolidol ratio when isoprenol is supplied is possibly a reflection of a higher IPP availability over DMAPP, favouring the formation of sesquiterpenoids over monoterpenoids. Next, isoprenol was replaced with prenol as substrate to increase the amount of DMAPP, and as such increase linalool levels over nerolidol (Figure 5B). Indeed, an increase in the linalool/nerolidol ratio was observed for all conditions, reaching a 1 : 1 proportion for most conditions using p(Iso)prenol‐GPPS‐bLinS. Approximately 25 mg L_org_^−1^ linalool was produced using the single‐plasmid system and 25 mM prenol. Linalool and nerolidol levels remained low for the two‐plasmid system. As for isoprenol, the addition of prenol at higher concentrations (37.5 and 50 mM) resulted in reduced product level (Figure 5B).

To further optimize secondary substrate supply, isoprenol and prenol were added combined at equal concentration as a tentative to mimic of the required IPP and DMAPP ratio to form GPP (Figure 5C). The combination of substrates had little influence on the product titres in the two‐plasmid system, but it further increased both linalool and nerolidol levels in the single‐plasmid system. In this case, linalool and nerolidol titres reached 55 mg L_org_^−1^ and 75 mg L_org_^−1^ respectively, using a combination of 12.5 mM isoprenol and 12.5 mM prenol. A higher concentration of these substrates combined significantly reduced monoterpenoid and sesquiterpenoid titres. These initial experiments validated the superior performance of the single‐plasmid system over the two‐plasmid system and the best substrate concentration of 12.5 mM isoprenol and prenol. These conditions were used for further optimization of linalool production.

**Figure 6:**
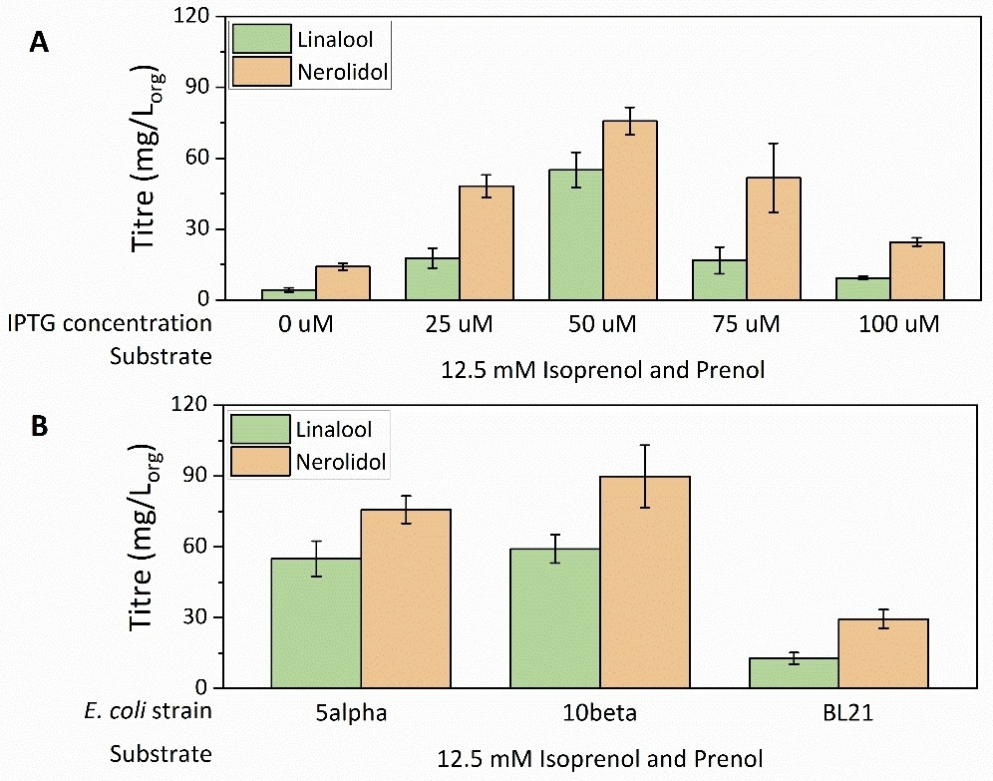
Linalool and nerolidol titres obtained with the IU pathway using the plasmid p(Iso)prenol‐GPPS‐bLinS, 12.5 mM isoprenol and prenol as substrates, with different IPTG concentrations (A), and different cell strains (B). *E. coli* cells were grown in 3 mL TB media with 20 % *n*‐nonane overlay for 72 h. Error bars represent the standard deviation of three biological replicates.

The influence of other parameters such as inducer concentration and *E. coli* strain was next evaluated for linalool production using the IU pathway. Inducer level is known to have a direct impact on the metabolic burden imposed on the cell, affecting growth rates, product levels, protein expression and plasmid stability.^[24]^ To determine the best inducer level, the isopropyl β‐D‐1‐thiogalactopyranoside (IPTG) concentration was varied from 0 to 100 μM, and linalool and nerolidol titres were determined. Within the IPTG concentration range tested, 50 μM gave the best results (Figure 6A).

Linalool production was evaluated using three different *E. coli* strains (NEB‐5α, NEB‐10β, and BL21) (Figure 6B). Similar to the results observed for the synthesis of larger terpenoids,^[25]^ linalool production had a significant strain dependence. NEB‐10β cells increased linalool production to almost 60 mg L_org_^−1^ and production of nerolidol to almost 90 mg L_org_^−1^ when 12.5 mM isoprenol and prenol was used as feedstock. Using BL21 cells, the monoterpenoid production decreased to 12 mg L_org_^−1^. This strain also showed lower linalool/nerolidol ratio, producing 29 mg L_org_^−1^ nerolidol. Although BL21 cells are widely used for protein expression, high gene expression can lead to the formation of protein aggregates or surplus synthesis of proteins in part, resulting in an adverse metabolic pathway balance.^[26]^

**Figure 7:**
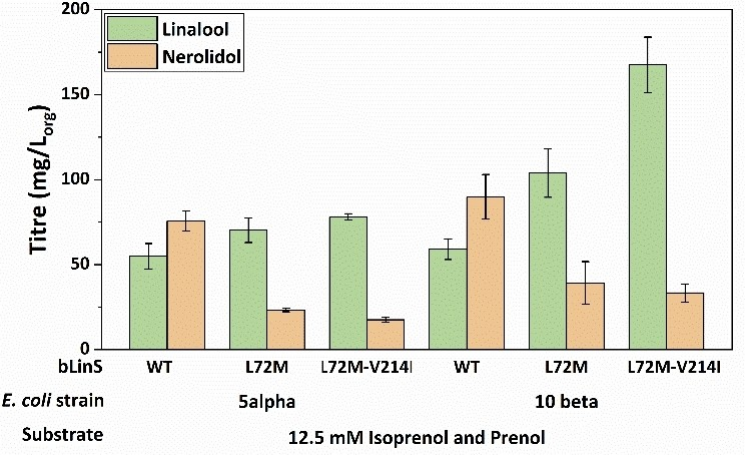
Linalool and nerolidol titres obtained with the IU pathway using the plasmid p(iso)prenol‐GPPS‐bLinS, 12.5 mM isoprenol and prenol as substrates, NEB‐5α and NEB‐10β cells and different bLinS variants. *E. coli* cells were grown in 3 mL TB media with 20 % *n*‐nonane overlay for 72 h. Error bars represent the standard deviation of three biological replicates.

Having identified residues that influence linalool and nerolidol production, the best performing bLinS variants, L72M and L72M‐V214I were tested in the IU pathway using the p(Iso)prenol‐GPPS‐bLinS plasmid. Linalool titres increased with both variants, reaching almost 80 mg L_org_^−1^ using the L72M‐V214I bLinS and NEB‐5α cells, and 167 mg L_org_^−1^ using L72M‐V214I bLinS and NEB‐10β cells, the highest linalool titre obtained using the IU pathway to date (Figure 7).

Crucially, for the first time nerolidol titres remained lower than linalool levels using the IU pathway, representing approximately 27 and 17 % of total terpenoids produced using the L72M and L72M‐V214I variants, respectively. Similar to what was observed with the MVA pathway, both bLinS variants increased linalool titres compared to wild type bLinS, but the double variant also increased the linalool/nerolidol ratio compared to the WT bLinS. Interestingly, these results are aligned to what was observed in the *in vitro* assay. Although both the MVA pathway and IU pathway are responsible for producing the IPP and DMAPP pool, which is used by prenyl transferases to elongate terpenoid chains, the higher relative amount of nerolidol from the IU pathway suggests a greater availability of FPP over GPP compared to the MVA pathway, even under optimised conditions.

**Figure 8:**
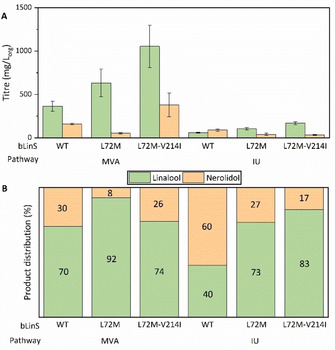
Comparison of optimised linalool and nerolidol product titres (A) and product ratio (B) obtained with the MVA pathway (NEB‐5α cells) and the IU pathway (NEB‐10β cells, 12.5 mM each isoprenol and prenol as substrates) using different bLinS variants.

Although linalool levels produced through the IU pathway were lower than the titres obtained with the MVA pathway (Figure 8), this should not eclipse its potential. The MVA pathway was first engineered in *E. coli* almost two decades ago, and the first terpenoid titres obtained were also on the level of a few dozens to a hundred mg/L.^[8b]^ Since then, numerous studies identified and optimized fermentation conditions, feedback‐resistant enzymes, accumulation of toxic intermediates, and other process and metabolic bottlenecks, allowing the increase of terpenoid titres. We report the use of IU pathway to produce terpenoids in *E. coli* for the second time and the monoterpenoid titres obtained are already superior to the ones obtained previously.^[14]^ The isoprenol and prenol utilizing pathways were only recently discovered, and little is known about how the enzymatic steps are regulated. A similar pathway developed by Clomburg *et al*. produced up to 2 g L^−1^ geranoids, showing that with further optimization it is possible to reach titres comparable to the ones achieved using the MVA pathway.^[13]^

The IU pathway strategy benefits from a reduced number of genes, which can lead to an accelerated and easier optimization process. Our changes in plasmid system, substrate and inducer concentration, and host cell strain increased linalool titre 280‐fold compared to initial conditions. Using an improved bLinS variant further tripled linalool titres and allowed formation of fewer by‐products resulting in a more pure product. A major advantage of the IU pathway over the MVA pathway is the ability to direct flux to GPP or FPP by balancing the prenol and isoprenol feedstock concentrations, resulting in a more versatile terpenoid production platform. The manipulation of other parameters using the metabolic engineering toolbox may further improve carbon flux towards the terpenoid of interest.

## Conclusion

Linalool is a monoterpenoid with applications ranging from cosmetic products to biofuels, and over half of total linalool consumed is produced through traditional chemical processes.^[27]^ In this study, we created bLinS variants which resulted in increased linalool and reduced nerolidol levels when expressed in engineered *E. coli* for the production of terpenoids. Mutations L72M and V214I/L were shown to act as gatekeepers for FPP acceptance by re‐shaping the active site. WT and bLinS variants were co‐expressed with the canonical MVA pathway and L72M‐V214I mutation in bLinS increased linalool titres almost 3‐fold, producing 1054 mg L_org_^−1^ linalool. Nerolidol titres corresponded to 26 % total terpenoid recovered and reached 379 mg L_org_^−1^.

We compared the MVA pathway to the non‐canonical IU pathway which uses isoprenol and prenol as secondary substrates to form the terpenoid building blocks IPP and DMAPP, through a reduced number of enzymatic steps. We balanced isoprenol and prenol concentrations to modulate linalool and nerolidol titres, and optimised plasmid system, inducer concentration and cell type. Insertion of the engineered bLinS variants into the improved IU pathway increased linalool titres 800‐fold to 167 mg L_org_^−1^ with a reduction in nerolidol levels to 17 % of total terpenoid products.

Even though the highly optimised canonical MVA pathway results in higher linalool titres, compared to the IU pathway, the latter offers a more flexible terpenoid production platform with potential for further improvement. Decoupling product synthesis from central metabolism can increase the metabolic flux to the desired product. The intracellular GPP/FPP ratio can be balanced to match the product of interest using different prenol/isoprenol concentrations, without the need for additional genes or enzyme optimisation. The fewer number of genes in the IU pathway minimises the accumulation of potentially toxic intermediates, and simplifies future optimisation steps.

In conclusion, our results show a promising new and simpler pathway to produce linalool, and it adds to other similar initiatives to produce other terpenoids from isoprenol and prenol. Our proof of principle study resulted in 800‐fold improved linalool titres using minimal engineering. There is still a lack of understating of how this pathway is regulated and the identity of bottlenecks within the pathway. Further pathway tuning will lead to elucidation of how the metabolic flux is directed and this will form the basis of further pathway optimization.

## Experimental Section

**Chemicals**: GPP and FPP substrates were synthesized from geraniol and farnesol respectively as described previously.^[28]^

**Bacterial strains and media**: All *E. coli* strains were routinely grown in Lysogeny Broth (LB) or on LB agar plates including antibiotic supplements as appropriate (carbenicillin, 100 μg mL^−1^; kanamycin, 50 μg mL^−1^). Mutagenesis and plasmid propagation was performed using *E. coli* Stellar cells (Clontech). Terpenoids production was performed in phosphate buffered TB using *E. coli* DH5α, DH10β and BL21 (NEB 5α, NEB 10β and BL21(DE3), New England Biolabs).

**Isoprenol and prenol toxicity**: OD_600_ measurements were performed to evaluate isoprenol and prenol toxicity on *E. coli* NEB‐5α cells at different concentrations. Cultures from frozen glycerol stocks were incubated in 20 mL TB medium and grown overnight at 37 °C and 180 rpm. Overnight cultures were diluted with 25 mL TB medium in a ratio of 1 : 100, and incubated at 37 °C and 180 rpm until OD_600_ reached 0.4. 160 μL of the culture was transferred to a 96‐well plate previously filled with 40 μL solution of TB medium and isoprenol and/or prenol at the desired concentration. The microplate was transferred to a BMG Labtech ClarioStar plate reader and incubated at 30 °C and 300 rpm. OD_600_ measurements were made at least in triplicate for each sample over 24 h. The range of concentration evaluated was from 3 to 100 mM of isoprenol, 3 to 100 mM of prenol, and 3 to 100 mM of isoprenol and prenol in a 1 : 1 ratio.

**Plasmid construction and site‐directed mutagenesis**: The pMVA‐GPPS‐bLinS plasmid was constructed by replacing the limonene synthase (LimS) in plasmid pJBEI6410^[29]^ with linalool synthase from *Streptomyces clavuligerus* (bLinS).^[17]^ The bLinS gene was amplified from plasmid pGPPSmTC/S38^[17]^ using primer pair LinS+6410‐Fw/Rv. The pJBEI6410 plasmid, except LimS, was amplified using primer pair pJ6410_Syn_open3‐Fw and pJ6410_Syn_open5_Rv (Table S2). The fragments were ligated using the In‐Fusion® HD Cloning method (TaKaRa) following the manufacturer's instructions.

Gene sequences encoding the Isopentenol utilization (IU) pathway developed by Chatzivasileiou *et al*.^[14]^ were designed, codon‐optimized for *E. coli*, and purchased from GeneArt, Thermo Fisher Scientific in a pET21b plasmid. ScCK‐AtIPK‐EcIDI genes (Table S1) were amplified from the pET21b plasmid and EcoRI restriction site was added by PCR using DNA polymerase CloneAmp™ HiFi PCR Premix (Clontech) and primers pET21b‐EcoRI_Fw, and pET21b_Rv (Table S2), according to manufacturer's recommendation. PCR product was digested with EcoRI and XhoI, and run on a 1 % agarose gel. The band corresponding to the ScCK‐AtIPK‐EcIDI fragment was gel extracted and purified. pMVA plasmid (Table S4) was digested with EcoRI and XhoI, and the band corresponding to plasmid vector was gel extracted and purified. Vector and the fragment were ligated to obtain the p(Iso)prenol plasmid using New England Biolabs Quick Ligation™ kit. The product of ligation was transformed into competent *E. coli* NEB 5α cells. p(Iso)prenol‐GPPS‐bLinS plasmid was obtained using In‐Fusion® HD Cloning method (TaKaRa). Plasmid vector was linearized by PCR from the pMVA‐GPPS‐bLinS plasmid (Table S4) with the primers pMVA‐prenol‐bLinS_V_Fw and pMVA‐prenol‐bLinS_V_Rv (Table S2), and the ScCK‐AtIPK‐EcIDI fragment was linearized by PCR from the p(Iso)prenol plasmid using the primers pMVA‐prenol‐bLinS_F_Fw and pMVA‐prenol‐bLinS_F_Rv (Table S2) according to manufacturer's recommendation. Mutations were introduced in bLinS using the QuikChange site‐directed mutagenesis method (Stratagene) using plasmid pGPPSmTC/S38, pET‐bLinS, or p(Iso)prenol‐GPPS‐bLinS encoding native bLinS^[17]^ as template. The oligonucleotides used are shown in Table S3. All plasmids were confirmed by standard Sanger sequencing.

**Terpenoid production in*****E. coli***: For terpenoid production, p(Iso)prenol‐GPPS‐bLinS or a pGPPSmTC/S plasmid (Table S4) harbouring native or a variant bLinS gene was co‐transformed with p(Iso)prenol or pMVA into *E. coli* DH5α and grown as described previously.^[19]^ Briefly, expression strains were inoculated at 37 °C in 3 mL TB supplemented with 0.4 % glucose and the appropriate antibiotics in glass screw capped vials. After 7 h, isoprenol and/or prenol were added accordingly together with inducers (50 μM IPTG and 25 nM anhydro‐tetracycline, unless otherwise indicated) and 20 % *n*‐nonane organic layer to capture the volatile terpenoid products. After 72 h at 30 °C, the *n*‐nonane overlay was collected, dried over anhydrous MgSO_4_ and mixed at a 1 : 1 ratio with ethyl acetate containing 0.01 % (v/v) *sec*‐butylbenzene or 0.1 % (v/v) limonene as internal standards for GC‐MS analysis.

**Expression and purification of bLinS variants**: *E. coli* ArcticExpress (DE3) cells were freshly transformed with a pET‐bLinS plasmid (Table S4), and induced with 0.1 mM IPTG for 16 h at 16 °C. The recombinant proteins were purified as described previously.^[17]^ Briefly, the cells were harvested and resuspended in buffer A (25 mM Tris pH 8.0, 150 mM NaCl, 1 mM DTT, 4 mM MgCl_2_ and 5 % (v/v) glycerol). The cells were lysed by sonication and the debris was removed by centrifugation. The supernatant was loaded onto a His‐Trap column (GE Healthcare) pre‐equilibrated with buffer A containing 25 mM imidazole. The column was washed with buffer A containing 25 mM imidazole, and the proteins were eluted by increasing the imidazole concentration to 100 mM and 500 mM. Fractions containing the purified proteins were pooled, concentrated and desalted using a PD10 column (BioRad) prior to storage at −80 °C.

***In vitro*****biotransformations**: All biotransformation reactions with purified enzyme were prepared in duplicate in buffer A in glass vials. For product profile determination, the reaction mixtures (0.25 mL) contained 0.4 mM GPP, or FPP and 1 μM purified enzyme, and a 100 % (v/v) *n*‐nonane layer was added to capture the volatile monoterpenoid products. The vials were incubated at 30 °C with gentle shaking for 5 min. The reaction was stopped by placing the vials on ice, followed by immediate vortexing for 30s. The *n*‐nonane overlay was collected, dried over anhydrous MgSO_4_ and mixed at a 1 : 1 ratio with ethyl acetate containing 0.01 % (v/v) *sec*‐butylbenzene as internal standard. The samples were analysed by GC‐MS.

**GC‐MS analysis**: The samples were injected onto an Agilent Technologies 7890B GC equipped with an Agilent Technologies 5977A MSD. The products were separated on a DB‐WAX column (30 m×0.32 mm i.d., 0.25 μM film thickness, Agilent Technologies). The injector temperature was set at 240 °C with a split ratio of 20 : 1 (1 μL injection). The carrier gas was helium with a flow rate of 1 mL min^−1^ and a pressure of 5.1 psi. The following oven program was used: 50 °C (1 min hold), ramp to 68 °C at 5 °C min^−1^ (2 min hold), and ramp to 230 °C at 25 °C min^−1^ (2 min hold). The ion source temperature of the mass spectrometer (MS) was set to 230 °C and spectra were recorded from m/z 50 to m/z 250. Compound identification was carried out using authentic standards and comparison to reference spectra in the NIST library of MS spectra and fragmentation patterns as described previously.^[19]^

## Conflict of interest

NGHL and NSS declare the following competing interest: Patent pending for the use of bLinS variants in the production of linalool using synthetic biology methods.

## Supporting information

As a service to our authors and readers, this journal provides supporting information supplied by the authors. Such materials are peer reviewed and may be re‐organized for online delivery, but are not copy‐edited or typeset. Technical support issues arising from supporting information (other than missing files) should be addressed to the authors.

Supplementary
