# Supplementary material for: Isopentenol Utilization Pathway for the Production of Linalool in Escherichia coli Using an Improved Bacterial Linalool/Nerolidol Synthase
Source: Chembiochem. 2021 May 25;22(13):2325–34. doi: 10.1002/cbic.202100110 (PMC8362072; doi:10.1002/cbic.202100110)
Supplement: Supplementary file 1 — Supplementary [file CBIC-22-2325-s001.pdf]

ChemBioChem

Supporting Information

Isopentenol Utilization Pathway for the Production of Linalool in *Escherichia coli* Using an Improved Bacterial Linalool/Nerolidol Synthase

Clara A. Ferraz, Nicole G. H. Leferink, Iaroslav Kosov, and Nigel S. Scrutton*

Table of Contents

EXPERIMENTAL SECTION 3

Table S1: Enzymes of the Isopentenol Utilization (IU) pathway 3

Table S2: Oligonucleotides used for plasmid construction. 3

Table S3: Oligonucleotides used for site-directed mutagenesis. 3

Table S4: Plasmids used in this study 5

RESULTS SECTION 7

Table S5: Summary of product profiles obtained using the IU pathway. 7

Table S6: Summary product profiles obtained for WT and bLinS variants. 9

Figure S1: GC-MS analysis of monoterpenoid production strain containing wild-type bLinS. 10

Figure S2: GC-MS analysis of terpenoid production strain containing the IU pathway with different concentrations of isoprenol. 11

Figure S3: GC-MS analysis of terpenoid production strain containing the IU pathway with different concentrations of prenol...12

Figure S4: GC-MS analysis of terpenoid production strain containing the IU pathway with different concentrations of isoprenol and prenol...13

Figure S5: GC-MS analysis of terpenoid production strain containing the IU pathway with different concentrations of IPTG, *E. coli* cell strain and bLinS mutant. 14

Figure S6: GC-MS analysis of first round bLinS variants...15

Figure S7: GC-MS analysis of second round bLinS variants...15

Figure S8: GC-MS analysis of bLinS-L72 variants. 16

Figure S9: GC-MS analysis of bLinS-V214 variants...16

Figure S10: GC-MS analysis bLinS variants with multiple mutations...17

Figure S11: Comparison of product profiles wild-type bLinS (WT) and first round variants. 18

Figure S12: Properties of best performing bLinS variants when expressed in engineered *E. coli* for terpenoid production..19

REFERENCES 20

EXPERIMENTAL SECTION

Table S1: Enzymes of the Isopentenol Utilization (IU) pathway

Origin organism	Enzyme	UniProt code
Saccharomyces cerevisiae	Choline Kinase (ScCK)	P20485
Arabidopsis thaliana	Isopentenyl Phosphate Kinase (AtIPK)	Q8H1F7
Eschericia coli	Isopentenyl-pyrophosphate delta-isomerase (EcIDI)	Q46822

Table S2: Oligonucleotides used for plasmid construction.

Primer name	Sequence (5' → 3')
LinS+6410_Fw	TCAGAATTAAGGATCTTTTAAGAAGGAGATATACATGCAGGAATTTGAATTTGCG
LinS+6410_Rv	TCCTTACTCGAGTTTGGATCCTTAACCGCTGCTACGTGCCA
pJ6410_Syn_open3_Fw	GGATCCAAACTCGAGTAAGGA
pJ6410_Syn_open5_Rv	CTTAAAAGATCCTTAATTCTGACGA
pET21b-EcoRI_Fw	TTAATTGAATTCAACGCAAGGAAACACATTAAGGAGG
pET21b_Rv	CTTCCTTTCGGGCTTTGTTAGCA
pMVA-prenol-bLinS_V_Fw	GGATCTAGGAGGTAATGATAATG
pMVA-prenol-bLinS_V_Rv	TTCCTCCTAAGATCTTTTGAATTC
pMVA-prenol-bLinS_F_Fw	AGATCTTAGGAGGAACCTCTAGAAACGCAAGGAAACACA
pMVA-prenol-bLinS_F_Rv	TTACCTCCTAGATCCATAGACCTCCTTAGTTTTACTTGCT

Table S3: Oligonucleotides used for site-directed mutagenesis. Changed codons are underlined and changed nucleotides are in lower case. Forward oligonucleotides shown only.

Mutation	Primer name	Sequence (5' → 3')
L72F	bLinS-L72F_fw	CTGTGTGTTGATGTTtTcGGTTGGACCTTTCTG
L72M	bLinS-L72M_fw	CTGTGTGTTGATGTTaTGGGTTGGACCTTTCTG
L72I	bLinS-L72I_fw	CTGTGTGTTGATGTTaTtGGTTGGACCTTTCTG
L72N	bLinS-L72N_fw	CTGTGTGTTGATGTTaccGGTTGGACCTTTCTG
L72Q	bLinS-L72Q_fw	CTGTGTGTTGATGTTCaGGGTTGGACCTTTCTG
L72C	bLinS-L72C_fw	CTGTGTGTTGATGTTtgcGGTTGGACCTTTCTG
L72S	bLinS-L72S_fw	CTGTGTGTTGATGTTagcGGTTGGACCTTTCTG
L72T	bLinS-L72T_fw	CTGTGTGTTGATGTTaccGGTTGGACCTTTCTG
L72V	bLinS-L72V_fw	CTGTGTGTTGATGTTgTtGGTTGGACCTTTC
L72A	bLinS-L72A_fw	CTGTGTGTTGATGTTgcGGGTTGGACCTTTC
L72G	bLinS-L72G_fw	CTGTGTGTTGATGTTggcGGTTGGACCTTTCTG
T75F	bLinS-T75F_fw	GATGTTCTGGGTTGGttCTTTCTGTTTGATGATC
T75M	bLinS-T75M_fw	GATGTTCTGGGTTGGAtgTTTCTGTTTGATGATC
I176F	bLinS-I176F_fw	GCTCGCCATCGTACCtTTTGTTGTCGTCCGC
C177F	bLinS-C177F_fw	CATCGTACCATTTGTTttCGTCCGCTGTTTG
C178F	bLinS-C178F_fw	CATCGTACCATTTGTTtTCGTCCGCTGTTTG
C178M	bLinS-C178M_fw	CATCGTACCATTTGTatgCGTCCGCTGTTTG
V214F	bLinS-V214F_fw	CCACCAGTGATGCAtTTATTGGTATGAATG
V214L	bLinS-V214L_fw	CCACCAGTGATGCAcTgATTGGTATGAATG
V214I	bLinS-V214I_fw	CCACCAGTGATGCAaTTATTGGTATGAATG
V214M	bLinS-V214M_fw	CCACCAGTGATGCAaTgATTGGTATGAATG
V214S	bLinS-V214S_fw	CCACCAGTGATGCAagTATTGGTATGAATG
V214C	bLinS-V214C_fw	CACCAGTGATGCAtgTATTGGTATGAATGAAC

V214N	bLinS-V214N_fw	CCACCAGTGATGCAaaTATTGGTATGAATGAACTG
V214Q	bLinS-V214Q_fw	CCACCAGTGATGCAcagATTGGTATGAATGAACTG
V214T	bLinS-V214T_fw	CCACCAGTGATGCAaccATTGGTATGAATGAACTG
V214A	bLinS-V214A_fw	CACCAGTGATGCAGcgATTGGTATGAATGAAC
V214G	bLinS-V214G_fw	CACCAGTGATGCAGgcATTGGTATGAATGAAC
F295W	bLinS-F295W_fw	GCACTGAGCGCATggTGTCGTGGTTATC
F295Y	bLinS-F295Y_fw	GCACTGAGCGCATaTTGTCGTGGTTATC

Table S4: Plasmids used in this study

Plasmid reference	Plasmid name	Description	Source
pMVA	pBbA5k-MTSAe-T1f-MBI(f)-T1002i	p15A, Kanr, PlacUV5, MTSA, T1, MBI-f, T1002	[4]
pJBEI6410	pBbA5a-MTSAe-T1f-MBI(f)-T1002iPtrc-trGPPS(co)-LimS	p15A, Ampr, PlacUV5, MTSA, T1, MBI-f, T1002, Ptrc, trAgGPPS(co), LimS	[1]
pMVA-GPPS-bLinS	pBbA5a-MTSAe-T1f-MBI(f)-T1002i-trAgGPPS(co)-bLinS	p15A, Ampr, PlacUV5, MTSA, T1, MBI-f, T1002, Ptrc, trAgGPPS(co)- bLinS	This study
p(Iso)prenol	pBbA5k-ScCK-AtIPK-EcIDI	p15A, Kanr, PlaUV5, ScCK-AtIPK-EcIDI	This study
p(Iso)prenol-GPPS-bLinS	pBbA5a-ScCK-AtIPK-EcIDI-trAgGPPS(co)-bLinS	p15A, Ampr, PlacUV5, trc, ScCK-AtIPK-EcIDI-trAgGPPS(co)- bLinS	This study
p(Iso)prenol-GPPS-bLinS-L72M	pBbA5a-ScCK-AtIPK-EcIDI-trAgGPPS(co)-bLinS-L72M	p15A, Ampr, PlacUV5, trc, ScCK-AtIPK-EcIDI-trAgGPPS(co)- bLinS-L72M	This study
p(Iso)prenol-GPPS-bLinS-L72M-V214I	pBbA5a-ScCK-AtIPK-EcIDI-trAgGPPS(co)-bLinS-L72M-V214I	p15A, Ampr, PlacUV5, trc, ScCK-AtIPK-EcIDI-trAgGPPS(co)- bLinS-L72M-V214I	This study
pGPPSmTC/S38	pBbB2a-trAgGPPS(co)- bLinS	pBBR, Ampr, Ptet, trAgGPPS(co)- bLinS	[2]
pGPPSmTC/S38-L72F	pBbB2a-trAgGPPS(co)- bLinS-L72F	pBBR, Ampr, Ptet, trAgGPPS(co)- bLinS-L72F	This study
pGPPSmTC/S38-L72I	pBbB2a-trAgGPPS(co)- bLinS-L72I	pBBR, Ampr, Ptet, trAgGPPS(co)- bLinS-L72I	This study
pGPPSmTC/S38-L72N	pBbB2a-trAgGPPS(co)- bLinS-L72N	pBBR, Ampr, Ptet, trAgGPPS(co)- bLinS-L72N	This study
pGPPSmTC/S38-L72Q	pBbB2a-trAgGPPS(co)- bLinS-L72Q	pBBR, Ampr, Ptet, trAgGPPS(co)- bLinS-L72Q	This study
pGPPSmTC/S38-L72C	pBbB2a-trAgGPPS(co)- bLinS-L72C	pBBR, Ampr, Ptet, trAgGPPS(co)- bLinS-L72C	This study
pGPPSmTC/S38-L72S	pBbB2a-trAgGPPS(co)- bLinS-L72S	pBBR, Ampr, Ptet, trAgGPPS(co)- bLinS-L72S	This study
pGPPSmTC/S38-L72T	pBbB2a-trAgGPPS(co)- bLinS-L72T	pBBR, Ampr, Ptet, trAgGPPS(co)- bLinS-L72T	This study
pGPPSmTC/S38-L72V	pBbB2a-trAgGPPS(co)- bLinS-L72V	pBBR, Ampr, Ptet, trAgGPPS(co)- bLinS-L72V	This study
pGPPSmTC/S38-L72A	pBbB2a-trAgGPPS(co)- bLinS-L72A	pBBR, Ampr, Ptet, trAgGPPS(co)- bLinS-L72A	This study
pGPPSmTC/S38-L72G	pBbB2a-trAgGPPS(co)- bLinS-L72G	pBBR, Ampr, Ptet, trAgGPPS(co)- bLinS-L72G	This study
pGPPSmTC/S38-T75F	pBbB2a-trAgGPPS(co)- bLinS-T75F	pBBR, Ampr, Ptet, trAgGPPS(co)- bLinS-T75F	This study
pGPPSmTC/S38-C178F	pBbB2a-trAgGPPS(co)- bLinS-C178F	pBBR, Ampr, Ptet, trAgGPPS(co)- bLinS-C178F	This study
pGPPSmTC/S38-I176F	pBbB2a-trAgGPPS(co)- bLinS-I176F	pBBR, Ampr, Ptet, trAgGPPS(co)- bLinS-I176F	This study
pGPPSmTC/S38-C177F	pBbB2a-trAgGPPS(co)- bLinS-C177F	pBBR, Ampr, Ptet, trAgGPPS(co)- bLinS-C177F	This study
pGPPSmTC/S38-V214F	pBbB2a-trAgGPPS(co)- bLinS-V214F	pBBR, Ampr, Ptet, trAgGPPS(co)- bLinS-V214F	This study
pGPPSmTC/S38-F295W	pBbB2a-trAgGPPS(co)- bLinS-F295W	pBBR, Ampr, Ptet, trAgGPPS(co)- bLinS-F295W	This study
pGPPSmTC/S38-L72M	pBbB2a-trAgGPPS(co)- bLinS-L72M	pBBR, Ampr, Ptet, trAgGPPS(co)- bLinS-L72M	This study
pGPPSmTC/S38-T75M	pBbB2a-trAgGPPS(co)- bLinS-T75M	pBBR, Ampr, Ptet, trAgGPPS(co)- bLinS-T75M	This study
pGPPSmTC/S38-C178M	pBbB2a-trAgGPPS(co)- bLinS-C178M	pBBR, Ampr, Ptet, trAgGPPS(co)- bLinS-C178M	This study
pGPPSmTC/S38-V214L	pBbB2a-trAgGPPS(co)- bLinS-V214L	pBBR, Ampr, Ptet, trAgGPPS(co)- bLinS-V214L	This study
pGPPSmTC/S38-V214I	pBbB2a-trAgGPPS(co)- bLinS-V214I	pBBR, Ampr, Ptet, trAgGPPS(co)- bLinS-V214I	This study
pGPPSmTC/S38-V214M	pBbB2a-trAgGPPS(co)- bLinS-V214M	pBBR, Ampr, Ptet, trAgGPPS(co)- bLinS-V214M	This study
pGPPSmTC/S38-V214S	pBbB2a-trAgGPPS(co)- bLinS-V214S	pBBR, Ampr, Ptet, trAgGPPS(co)- bLinS-V214S	This study
pGPPSmTC/S38-V214C	pBbB2a-trAgGPPS(co)- bLinS-V214C	pBBR, Ampr, Ptet, trAgGPPS(co)- bLinS-V214C	This study
pGPPSmTC/S38-V214N	pBbB2a-trAgGPPS(co)- bLinS-V214N	pBBR, Ampr, Ptet, trAgGPPS(co)- bLinS-V214N	This study
pGPPSmTC/S38-V214Q	pBbB2a-trAgGPPS(co)- bLinS-V214Q	pBBR, Ampr, Ptet, trAgGPPS(co)- bLinS-V214Q	This study
pGPPSmTC/S38-V214T	pBbB2a-trAgGPPS(co)- bLinS-V214T	pBBR, Ampr, Ptet, trAgGPPS(co)- bLinS-V214T	This study
pGPPSmTC/S38-V214A	pBbB2a-trAgGPPS(co)- bLinS-V214A	pBBR, Ampr, Ptet, trAgGPPS(co)- bLinS-V214A	This study
pGPPSmTC/S38-V214G	pBbB2a-trAgGPPS(co)- bLinS-V214G	pBBR, Ampr, Ptet, trAgGPPS(co)- bLinS-V214G	This study

pGPPSmTC/S38-F295Y	pBbB2a-trAgGPPS(co)- bLinS-F295Y	pBBR, Ampr, Ptet, trAgGPPS(co)- bLinS-F295Y	This study
pGPPSmTC/S38-F295W	pBbB2a-trAgGPPS(co)- bLinS-F295W	pBBR, Ampr, Ptet, trAgGPPS(co)- bLinS-F295W	This study
pGPPSmTC/S38-L72F-C177F	pBbB2a-trAgGPPS(co)- bLinS-L72F-C177F	pBBR, Ampr, Ptet, trAgGPPS(co)- bLinS-L72F-C177F	This study
pGPPSmTC/S38-T75F-C177F	pBbB2a-trAgGPPS(co)- bLinS-T75F-C177F	pBBR, Ampr, Ptet, trAgGPPS(co)- bLinS-T75F-C177F	This study
pGPPSmTC/S38-L72F-T75-C177F	pBbB2a-trAgGPPS(co)- bLinS-L72F-T75-C177F	pBBR, Ampr, Ptet, trAgGPPS(co)- bLinS-L72F-T75-C177F	This study
pGPPSmTC/S38-L72M-V214I	pBbB2a-trAgGPPS(co)- bLinS-L72M-V214I	pBBR, Ampr, Ptet, trAgGPPS(co)- bLinS-L72M-V214I	This study
pGPPSmTC/S38-L72M-V214L	pBbB2a-trAgGPPS(co)- bLinS-L72M-V214L	pBBR, Ampr, Ptet, trAgGPPS(co)- bLinS-L72M-V214L	This study
pET-bLinS	pET-24d-His6-TEV-bLinS	pBR322, Kanr, T7, His6-TEV-bLinS	[2]
pET-bLinS-L72M	pET-24d-His6-TEV-bLinS-L72M	pBR322, Kanr, T7, His6-TEV-bLinS-L72M	This study
pET-bLinS-V214I	pET-24d-His6-TEV-bLinS-V214I	pBR322, Kanr, T7, His6-TEV-bLinS-V214I	This study
pET-bLinS-V214L	pET-24d-His6-TEV-bLinS-V214L	pBR322, Kanr, T7, His6-TEV-bLinS-V214L	This study
pET-bLinS-L72M-V214I	pET-24d-His6-TEV-bLinS-L72M-V214I	pBR322, Kanr, T7, His6-TEV-bLinS-L72M-V214I	This study
pET-bLinS-L72M-V214L	pET-24d-His6-TEV-bLinS-L72M-V214L	pBR322, Kanr, T7, His6-TEV-bLinS-L72M-V214L	This study

RESULTS SECTION

Table S5: Summary of product profiles obtained using the IU pathway. Product profiles and monoterpenoid titres (mg L_{org}^{-1}) were determined from two-phase cultures with an *n*-nonane overlay for each *E. coli* strain containing the IU pathway and a linalool producing module. Averages of 3 biological replicates and the corresponding standard deviations are shown.

Plasmid	Substrate	Inducer	*E. coli* strain	Linalool	Nerolidol	Reference
p(Iso)prenol + pGPPS-bLinS	7.5 mM isoprenol	50 µM IPTG, 2.5 µg/mL aTet	NEB 5α	0.14 ± 0.02	1.1 ± 0.6	This study
p(Iso)prenol + pGPPS-bLinS	12.5 mM isoprenol	50 µM IPTG, 2.5 µg/mL aTet	NEB 5α	0.21 ± 0.02	0.7 ± 0.2	This study
p(Iso)prenol + pGPPS-bLinS	25 mM isoprenol	50 µM IPTG, 2.5 µg/mL aTet	NEB 5α	0.22 ± 0.00	11.8 ± 1.0	This study
p(Iso)prenol + pGPPS-bLinS	37.5 mM isoprenol	50 µM IPTG, 2.5 µg/mL aTet	NEB 5α	0.20 ± 0.02	10.1 ± 1.4	This study
p(Iso)prenol + pGPPS-bLinS	50 mM isoprenol	50 µM IPTG, 2.5 µg/mL aTet	NEB 5α	0.14 ± 0.00	8.8 ± 1.4	This study
p(Iso)prenol + pGPPS-bLinS	7.5 mM prenol	50 µM IPTG, 2.5 µg/mL aTet	NEB 5α	0.44 ± 0.32	1.8 ± 1.3	This study
p(Iso)prenol + pGPPS-bLinS	12.5 mM prenol	50 µM IPTG, 2.5 µg/mL aTet	NEB 5α	2.08 ± 0.44	9.4 ± 1.7	This study
p(Iso)prenol + pGPPS-bLinS	25 mM prenol	50 µM IPTG, 2.5 µg/mL aTet	NEB 5α	2.58 ± 0.05	8.2 ± 0.4	This study
p(Iso)prenol + pGPPS-bLinS	37.5 mM prenol	50 µM IPTG, 2.5 µg/mL aTet	NEB 5α	1.98 ± 0.29	6.8 ± 0.9	This study
p(Iso)prenol + pGPPS-bLinS	50 mM prenol	50 µM IPTG, 2.5 µg/mL aTet	NEB 5α	0.91 ± 0.03	3.3 ± 0.6	This study
p(Iso)prenol + pGPPS-bLinS	3 mM isoprenol and 3 mM prenol	50 µM IPTG, 2.5 µg/mL aTet	NEB 5α	0.58 ± 0.27	3.2 ± 2.4	This study
p(Iso)prenol + pGPPS-bLinS	7.5 mM isoprenol and 7.5 mM prenol	50 µM IPTG, 2.5 µg/mL aTet	NEB 5α	1.00 ± 0.52	3.7 ± 0.7	This study
p(Iso)prenol + pGPPS-bLinS	12.5 mM isoprenol and 12.5 mM prenol	50 µM IPTG, 2.5 µg/mL aTet	NEB 5α	1.389± 0.38	7.3 ± 0.8	This study
p(Iso)prenol + pGPPS-bLinS	25 mM isoprenol and 25 mM prenol	50 µM IPTG, 2.5 µg/mL aTet	NEB 5α	0.52 ± 0.07	4.2 ± 0.4	This study
p(Iso)prenol + pGPPS-bLinS	37.5 mM isoprenol and 37.5 mM prenol	50 µM IPTG, 2.5 µg/mL aTet	NEB 5α	0.39 ± 0.07	2.0 ± 0.4	This study
p(Iso)prenol-GPPS-bLinS	7.5 mM isoprenol	50 µM IPTG	NEB 5α	5.86 ± 0.85	16. ± 2.1	This study
p(Iso)prenol-GPPS-bLinS	12.5 mM isoprenol	50 µM IPTG	NEB 5α	16.90 ± 1.76	42.3 ± 8.0	This study
p(Iso)prenol-GPPS-bLinS	25 mM isoprenol	50 µM IPTG	NEB 5α	7.68 ± 1.40	32.5 ± 2.1	This study
p(Iso)prenol-GPPS-bLinS	37.5 mM isoprenol	50 µM IPTG	NEB 5α	3.00 ± 0.69	14.4 ± 2.6	This study
p(Iso)prenol-GPPS-bLinS	50 mM isoprenol	50 µM IPTG	NEB 5α	1.81 ± 0.32	9.1 ± 1.0	This study
p(Iso)prenol-GPPS-bLinS	7.5 mM prenol	50 µM IPTG	NEB 5α	19.29 ± 0.45	19.7 ± 1.0	This study
p(Iso)prenol-GPPS-bLinS	12.5 mM prenol	50 µM IPTG	NEB 5α	21.97 ± 2.43	23.8 ± 2.0	This study
p(Iso)prenol-GPPS-bLinS	25 mM prenol	50 µM IPTG	NEB 5α	24.96 ± 1.23	25.9 ± 3.7	This study
p(Iso)prenol-GPPS-bLinS	37.5 mM prenol	50 µM IPTG	NEB 5α	15.26 ± 1.38	28.5 ± 2.7	This study
p(Iso)prenol-GPPS-bLinS	50 mM prenol	50 µM IPTG	NEB 5α	11.60 ± 1.20	20.7 ± 3.0	This study
p(Iso)prenol-GPPS-bLinS	3 mM isoprenol and 3 mM prenol	50 µM IPTG	NEB 5α	12.77 ± 2.73	18.7 ± 3.1	This study
p(Iso)prenol-GPPS-bLinS	7.5 mM isoprenol and 7.5 mM prenol	50 µM IPTG	NEB 5α	44.98 ± 3.29	63.5 ± 5.5	This study
p(Iso)prenol-GPPS-bLinS	12.5 mM isoprenol and 12.5 mM prenol	50 µM IPTG	NEB 5α	54.99 ± 7.51	75.7 ± 5.8	This study
p(Iso)prenol-GPPS-bLinS	25 mM isoprenol and 25 mM prenol	50 µM IPTG	NEB 5α	6.64 ± 0.36	24.0 ± 4.2	This study
p(Iso)prenol-GPPS-bLinS	37.5 mM isoprenol and 37.5 mM prenol	50 µM IPTG	NEB 5α	2.33 ± 0.51	9.4 ± 1.2	This study
p(Iso)prenol-GPPS-bLinS	12.5 mM isoprenol and 12.5 prenol	0 µM IPTG	NEB 5α	4.23 ± 0.94	14.0 ± 1.4	This study
p(Iso)prenol-GPPS-bLinS	12.5 mM isoprenol and 12.5 prenol	25 µM IPTG	NEB 5α	17.68 ± 4.22	48.2 ± 4.8	This study
p(Iso)prenol-GPPS-bLinS	12.5 mM isoprenol and 12.5 prenol	75 µM IPTG	NEB 5α	16.71 ± 5.67	51.7 ± 14.6	This study

p(Iso)prenol-GPPS-bLinS	12.5 mM isoprenol and 12.5 prenol	100 µM IPTG	NEB 5α	9.29 ± 0.71	24.5 ± 1.7	This study
p(Iso)prenol-GPPS-bLinS	12.5 mM isoprenol and 12.5 mM prenol	50 µM IPTG	NEB 10 β	59.14 ± 6.00	89.9 ± 13.2	This study
p(Iso)prenol-GPPS-bLinS	12.5 mM isoprenol and 12.5 mM prenol	50 µM IPTG	BL 21	12.79 ± 2.49	29.5 ± 3.9	This study
p(Iso)prenol-GPPS-bLinS-L72M	12.5 mM isoprenol and 12.5 mM prenol	50 µM IPTG	NEB 5α	70.30 ± 7.22	23.4 ± 1.1	This study
p(Iso)prenol-GPPS-bLinS-L72M-V214I	12.5 mM isoprenol and 12.5 mM prenol	50 µM IPTG	NEB 5α	78.22 ± 1.86	17.6 ± 1.5	This study
p(Iso)prenol-GPPS-bLinS-L72M	12.5 mM isoprenol and 12.5 mM prenol	50 µM IPTG	NEB 10 β	103.91 ± 14.23	39.3 ± 12.5	This study
p(Iso)prenol-GPPS-bLinS-L72M-V214I	12.5 mM isoprenol and 12.5 mM prenol	50 µM IPTG	NEB 10 β	167.61 ± 16.36	33.4 ± 5.3	This study

Table S6: Summary product profiles obtained for WT and bLinS variants. Product profiles and monoterpenoid titres (mg L_{org}^{-1}) were determined from two-phase cultures with an *n*-nonane overlay for each *E. coli* strain containing the MVA pathway and a bLinS variant. Averages of 2-6 biological replicates and the corresponding standard deviations are shown. ND = Not detected.

Variant	Linalool	Nerolidol	Geranoids	Farnesol[a]	Reference
WT bLinS	363.3 ± 57.9	159.1 ± 7.9	36.7 ± 0.8	31.5 ± 3.7	[2]
bLinS-L72F	2.8 ± 0.2	ND	31.8 ± 9.1	8.3 ± 9.4	This study
bLinS-L72M	632.7 ± 158.3	51.7 ± 8.9	9.7 ± 10.7	6.1 ± 2.4	This study
bLinS-L72I	1.1 ± 0.2	ND	13.9 ± 6.3	1.7 ± 2.0	This study
bLinS-L72N	0.03 ± 0.05	ND	20.9 ± 12.3	5.7 ± 2.8	This study
bLinS-L72Q	0.2 ± 0.1	ND	19.1 ± 7.1	1.7 ± 0.2	This study
bLinS-L72C	31.9 ± 58.2	14.1 ± 27.3	73.2 ± 102.7	3.2 ± 3.8	This study
bLinS-L72S	0.6 ± 0.4	0.4 ± 0.4	45.4 ± 23.1	0.5 ± 0.7	This study
bLinS-L72T	2.7 ± 1.8	6.4 ± 5.0	75.6 ± 72.0	10.6 ± 11.1	This study
bLinS-L72V	10.7 ± 11.2	9.5 ± 11.0	83.8 ± 84.1	3.8 ± 3.8	This study
bLinS-L72A	1.8 ± 1.3	13.0 ± 22.5	87.9 ± 48.9	10.4 ± 14.5	This study
bLinS-L72G	1.7 ± 0.2	0.8 ± 0.1	92.7 ± 11.0	2.4 ± 0.7	This study
bLinS-T75F	ND	ND	40.8 ± 14.1	15.5 ± 11.1	This study
bLinS-T75M	1.7 ± 1.2	0.1 ± 0.2	54.3 ± 24.0	2.3 ± 1.5	This study
bLinS-I176F	ND	ND	17.2 ± 16.8	17.2 ± 8.2	This study
bLinS-C177F	ND	ND	35.3 ± 9.4	9.2 ± 2.4	This study
bLinS-C178F	ND	ND	44.5 ± 14.8	29.8 ± 16.3	This study
bLinS-C178M	4.8 ± 0.1	0.5 ± 0.0	55.9 ± 15.7	1.4 ± 0.2	This study
bLinS-V214F	0.1 ± 0.1	ND	24.3 ± 24.0	5.8 ± 5.3	This study
bLinS-V214L	49.2 ± 4.8	2.1 ± 0.8	79.1 ± 11.6	2.0 ± 0.4	This study
bLinS-V214I	265.3 ± 39.2	109.7 ± 52.1	6.8 ± 1.5	9.3 ± 10.8	This study
bLinS-V214M	0.9 ± 0.7	ND	6.1 ± 6.7	1.7 ± 1.8	This study
bLinS-V214S	0.2 ± 0.1	ND	21.8 ± 9.1	1.8 ± 0.9	This study
bLinS-V214C	40.5 ± 73.5	3.3 ± 6.3	58.5 ± 74.5	5.6 ± 4.0	This study
bLinS-V214N	0.4 ± 0.1	ND	62.1 ± 37.3	4.8 ± 37.3	This study
bLinS-V214Q	0.6 ± 0.2	ND	105.3 ± 6.2	5.9 ± 2.1	This study
bLinS-V214T	0.5 ± 0.2	ND	41.4 ± 14.0	3.0 ± 2.1	This study
bLinS-V214A	0.6 ± 0.1	ND	57.9 ± 5.3	2.4 ± 0.9	This study
bLinS-V214G	0.2 ± 0.1	ND	28.6 ± 18.1	4.2 ± 0.6	This study
bLinS-F295W	0.3 ± 0.6	ND	26.9 ± 11.3	6.6 ± 5.6	This study
bLinS-F295Y	1.4 ± 0.5	0.6 ± 0.3	51.4 ± 23.3	1.5 ± 1.1	This study
bLinS-L72F-C177F	ND	ND	14.1 ± 12.2	15.7 ± 8.9	This study
bLinS-T75F-C177F	ND	ND	72.6 ± 33.2	31.9 ± 21.4	This study
bLinS-L72F-T75F-C177F	ND	ND	63.6 ± 12.6	18.9 ± 1.9	This study
bLinS-L72M-V214I	1054.0 ± 245.2	379.0 ± 134.3	13.1 ± 4.2	9.9 ± 5.2	This study
bLinS-L72M-V214L	192.6 ± 86.5	5.8 ± 3.5	38.4 ± 22.4	21.9 ± 30.2	This study

[a] Farnesol is a sesquiterpene by-product from endogenous *E. coli* activity[4-5], thus the lack of nerolidol production is not the result of the absence of FPP substrate.

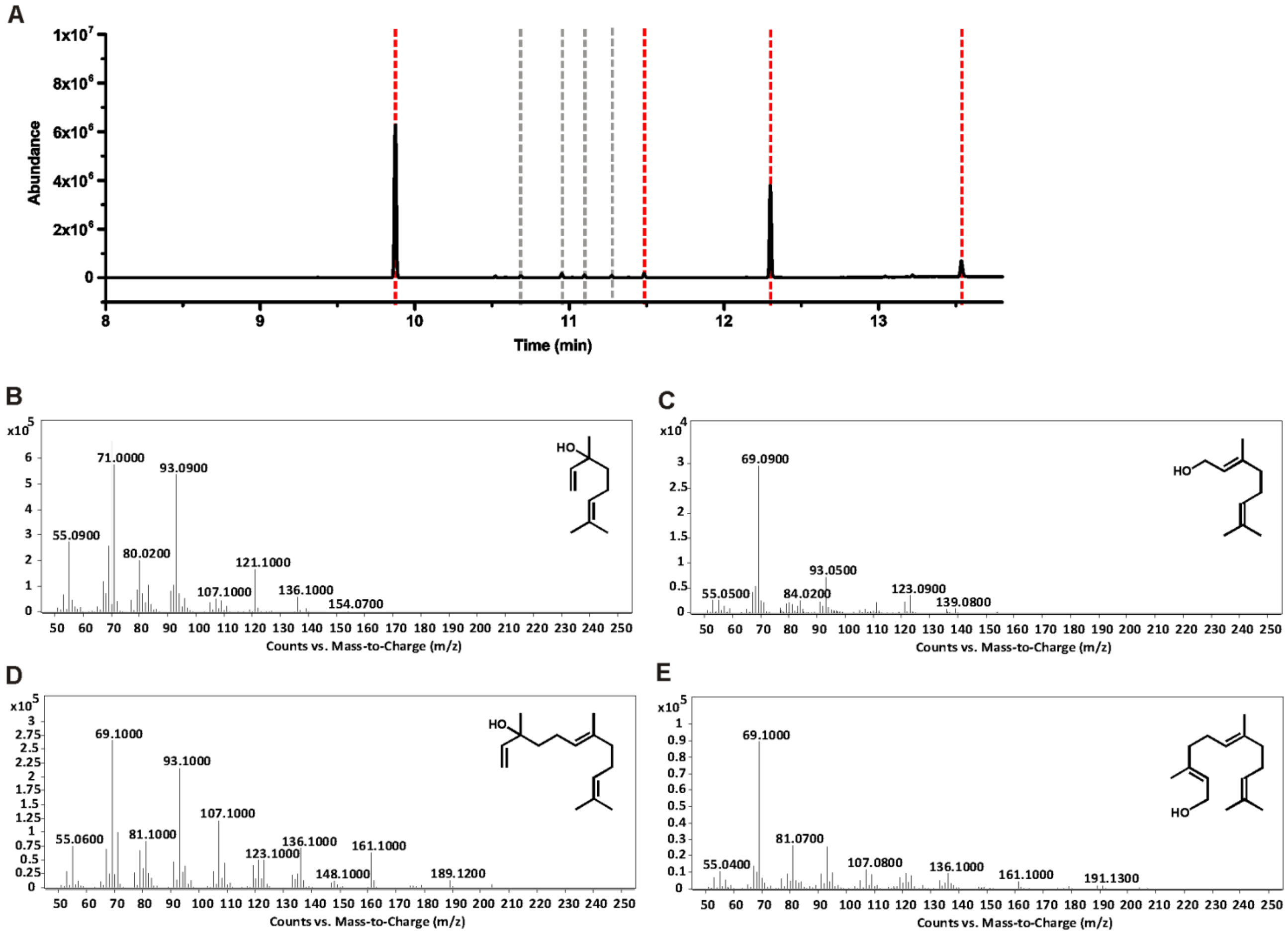

Figure S1: GC-MS analysis of monoterpenoid production strain containing wild-type bLinS. (**A**) Total ion count chromatogram of wild-type bLinS. Monoterpenoid peaks indicated with dashed red lines are: linalool (rt: 9.8 min); geraniol (rt: 11.4 min); *trans*-nerolidol (rt: 12.3 min); and farnesol (rt: 13.5 min). Compounds indicated with a dashed grey line are geraniol derivatives detected due to endogenous *E. coli* activity[4], and include neral (rt: 10.6 min), geranial (rt: 10.9 min), citronellol (rt: 11.1 min), and nerol (rt: 11.3 min). (**B**) MS spectrum of linalool. (**C**) MS spectrum of geraniol. (**D**) MS spectrum of *trans*-nerolidol. (**E**) MS spectrum of farnesol. Data obtained from Karuppiah et al 2017[2].

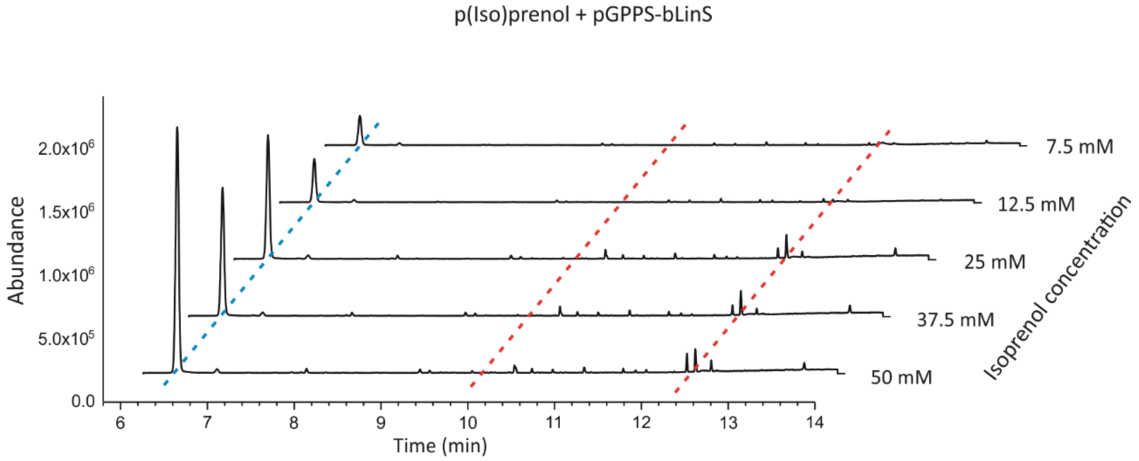

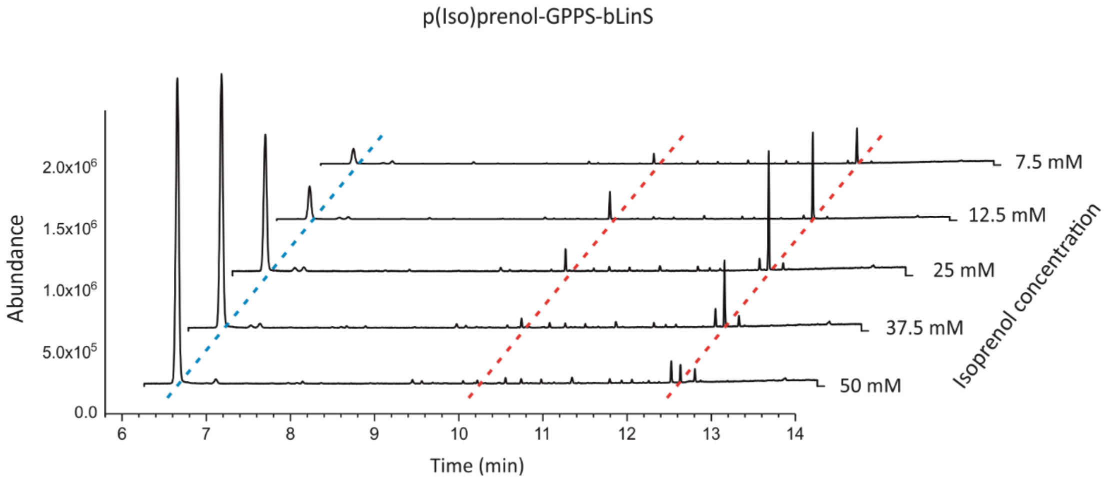

Figure S2: GC-MS analysis of terpenoid production strain containing the IU pathway with different concentrations of isoprenol. Representative total ion count chromatograms of IU pathway with the GPPS-bLinS module in the two-plasmid system and one-plasmid system expressed in *E. coli* NEB-5α. Isoprenol peak is indicated with dashed blue line (rt: 6.4 min) and terpenoid peaks indicated with dashed red lines are linalool (rt: 9.9 min) and nerolidol (rt: 12.3 min).

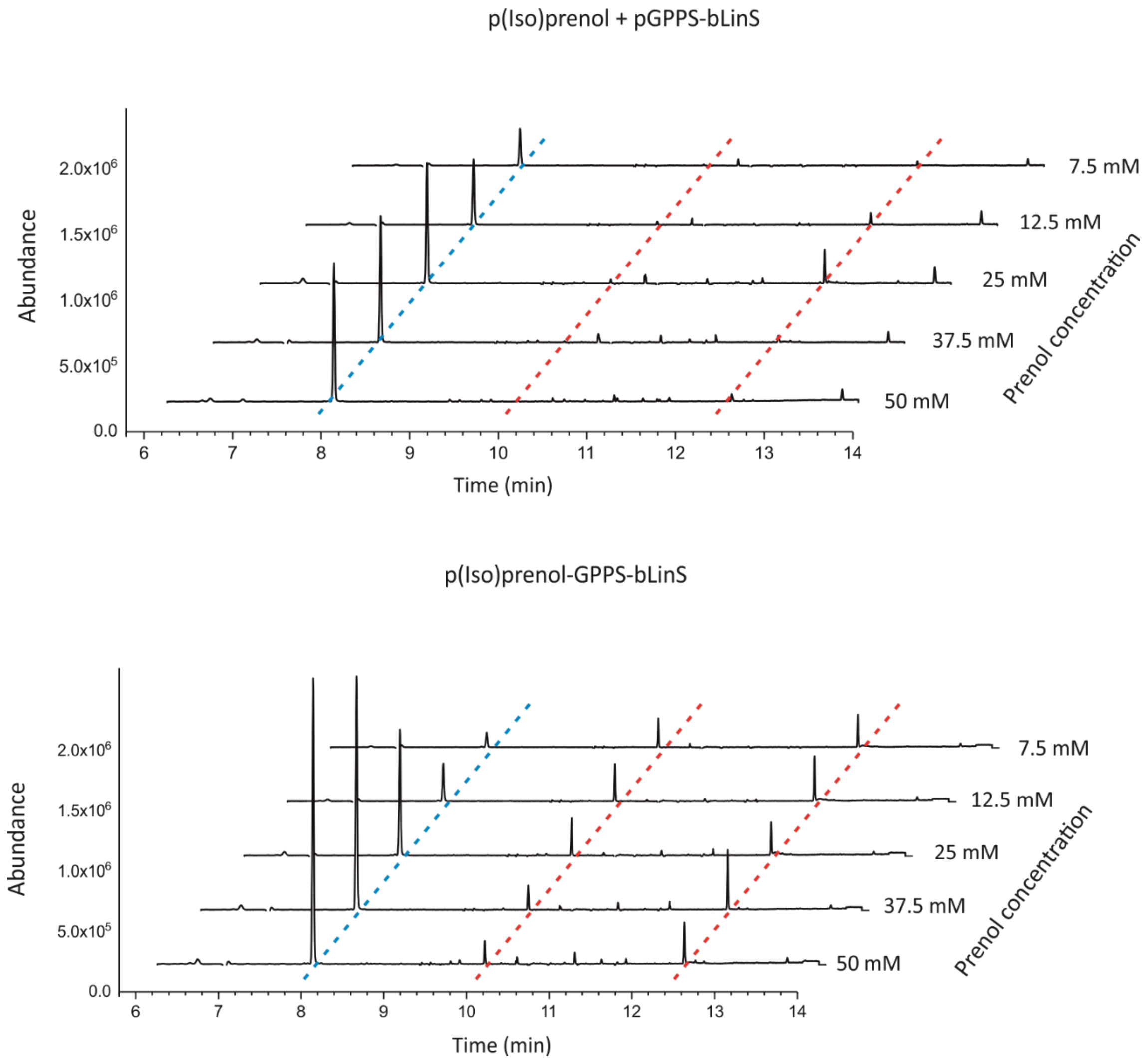

Figure S3: GC-MS analysis of terpenoid production strain containing the IU pathway with different concentrations of prenol. Representative total ion count chromatograms of IU pathway with the GPPS-bLinS module in the two plasmid system and one plasmid system expressed in *E. coli* NEB-5α. Prenol peak is indicated with a dashed blue line (rt: 7.9 min) and terpenoid peaks indicated with dashed red lines are linalool (rt: 9.9 min) and nerolidol (rt: 12.3 min).

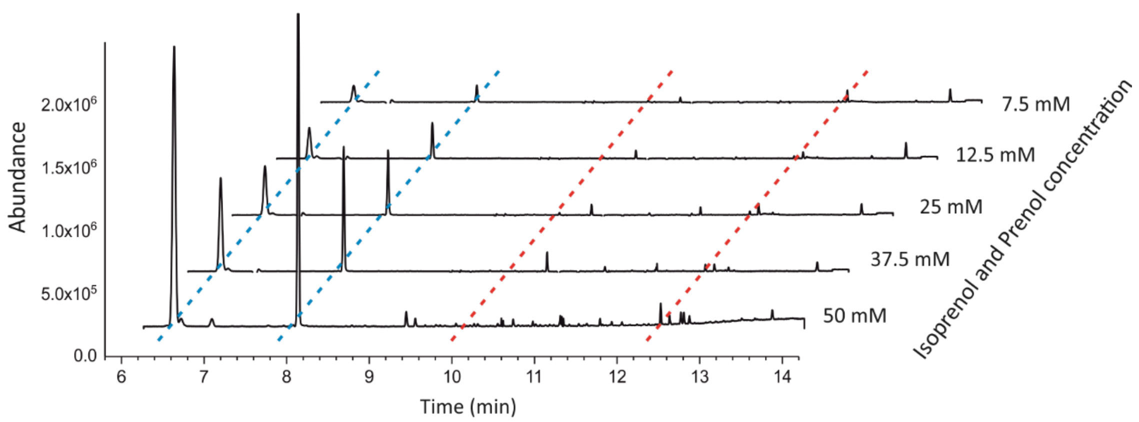

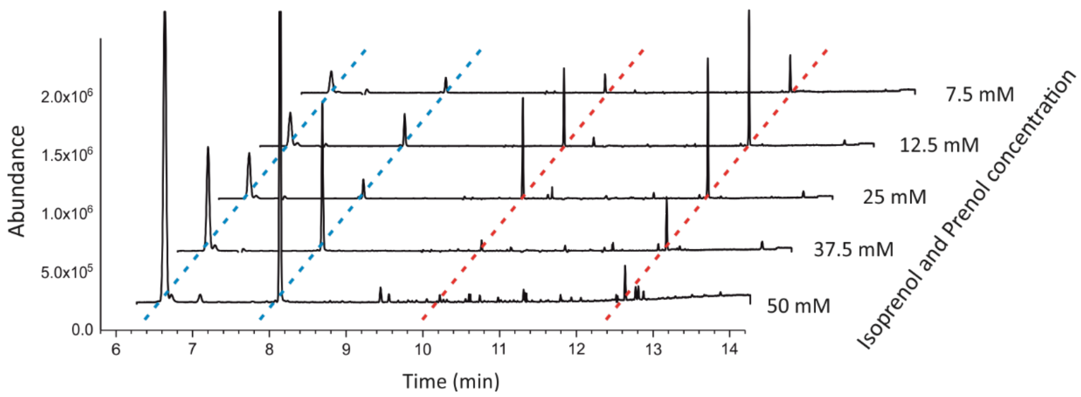

Figure S4: GC-MS analysis of terpenoid production strain containing the IU pathway with different concentrations of isoprenol and prenol. Representative total ion count chromatograms of IU pathway with the GPPS-bLinS module in the two-plasmid system and one-plasmid system expressed in *E. coli* NEB-5α. Isoprenol and prenol peaks are indicated with a dashed blue lines (rt: 6.4 and 7.9 min, respectively) and terpenoid peaks indicated with dashed red lines are linalool (rt: 9.9 min) and nerolidol (rt: 12.3 min).

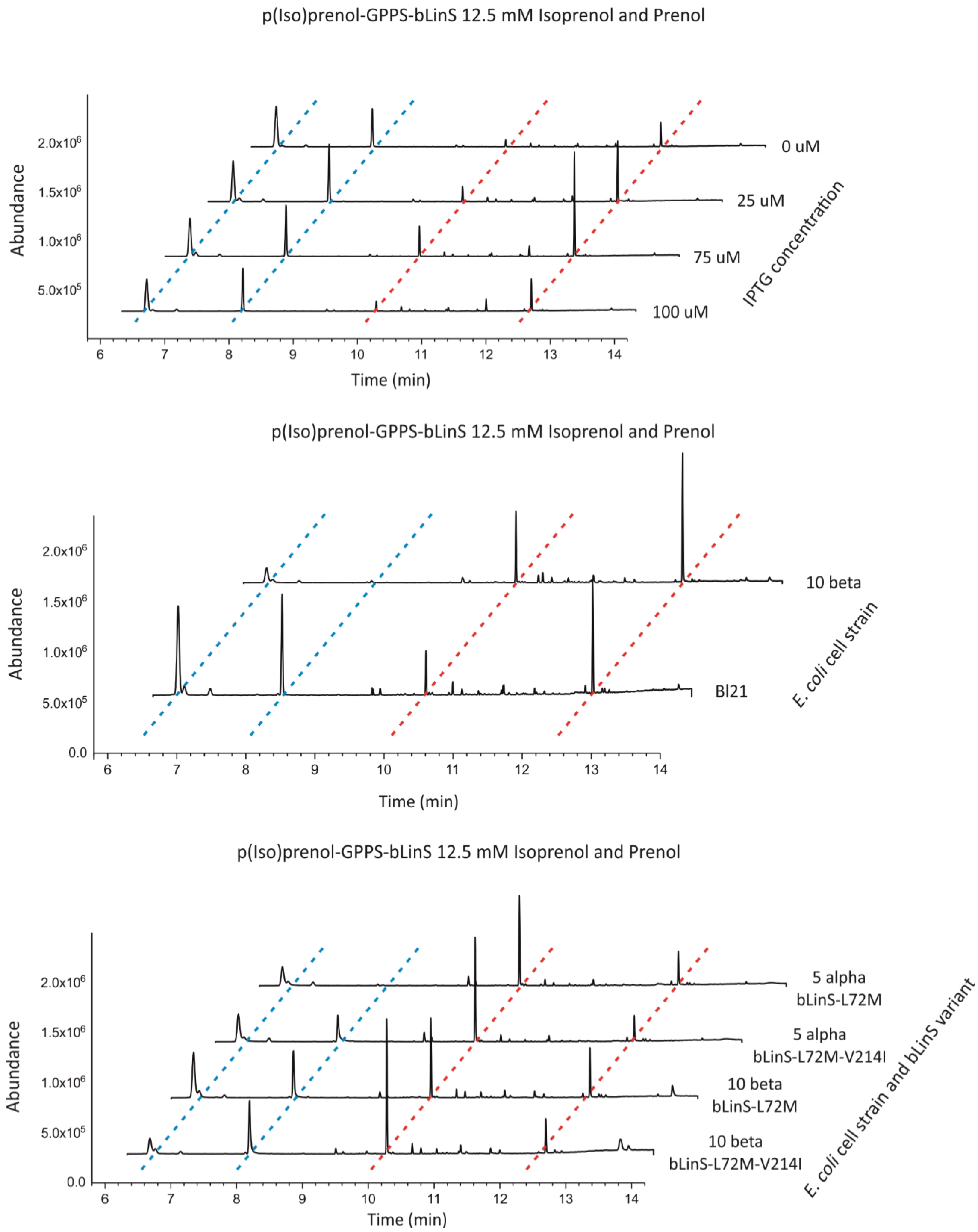

Figure S5: GC-MS analysis of terpenoid production strain containing the IU pathway with different concentrations of IPTG, *E. coli* cell strain and bLinS mutant. Representative total ion count chromatograms of IU pathway with the GPPS-bLinS module in the single-plasmid system expressed in *E. coli*. Isoprenol and prenol peaks are indicated with a dashed blue lines (rt: 6.4 and 7.9 min, respectively). Terpenoid peaks indicated with dashed red lines are linalool (rt: 9.9 min) and nerolidol (rt: 12.3 min).

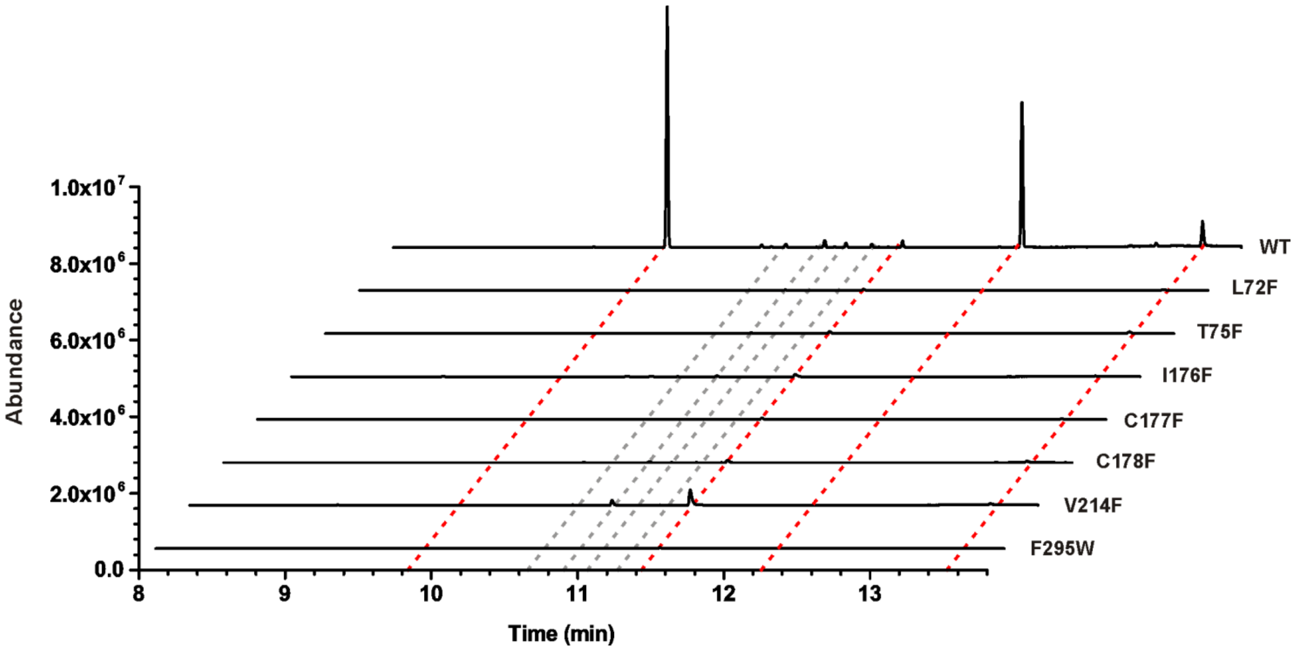

Figure S6: GC-MS analysis of first round bLinS variants. Representative total ion count chromatograms of wild-type bLinS, bLinS-L72F, bLinS-T75F, bLinS-I176F, bLinS-C177F, bLinS-C178F, bLinS-V214F, and bLinS-F295W when expressed in the *E. coli* monoterpenoid production platform[4]. Monoterpenoid peaks indicated with dashed red lines are: linalool (rt: 9.8 min), geraniol (rt: 11.4 min), *trans*-nerolidol (rt: 12.3 min), and farnesol (rt: 13.5 min). Compounds indicated with a dashed grey line are geraniol derivatives detected due to endogenous *E. coli* activity[4], and include neral (rt: 10.6 min), geranial (rt: 10.9 min), citronellol (rt: 11.1 min), and nerol (rt: 11.3 min).

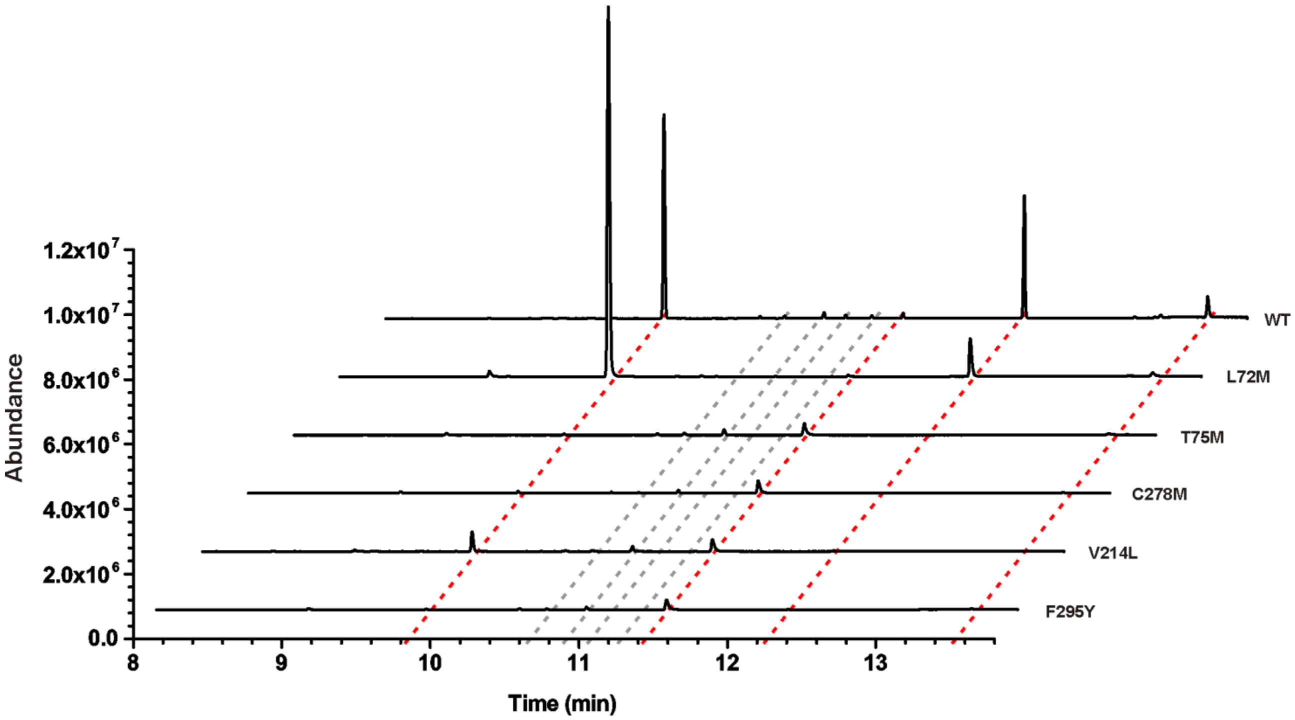

Figure S7: GC-MS analysis of second round bLinS variants. Representative total ion count chromatograms of wild-type bLinS, bLinS-L72M, bLinS-T75M, bLinS-C178M, bLinS-V214L, and bLinS-F295Y when expressed in the *E. coli* monoterpenoid production platform[4]. Monoterpenoid peaks indicated with dashed red lines are: linalool (rt: 9.8 min), geraniol (rt: 11.4 min), *trans*-nerolidol (rt: 12.3 min), and farnesol (rt: 13.5 min). Compounds indicated with a dashed grey line are geraniol derivatives detected due to endogenous *E. coli* activity[4], and include neral (rt: 10.6 min), geranial (rt: 10.9 min), citronellol (rt: 11.1 min), and nerol (rt: 11.3 min).

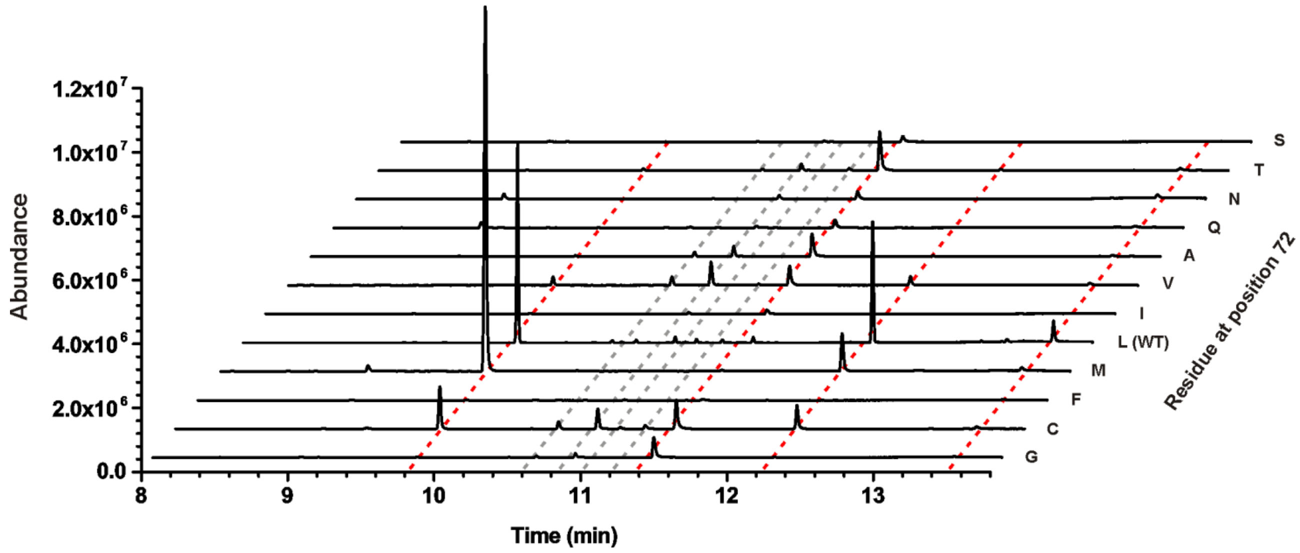

Figure S8: GC-MS analysis of bLinS-L72 variants. Representative total ion count chromatograms of bLinS-L72 variants when expressed in the *E. coli* monoterpenoid production platform[4]. Monoterpenoid peaks indicated with dashed red lines are: linalool (rt: 9.8 min), geraniol (rt: 11.4 min), *trans*-nerolidol (rt: 12.3 min), and farnesol (rt: 13.5 min). Compounds indicated with a dashed grey line are geraniol derivatives detected due to endogenous *E. coli* activity[4], and include neral (rt: 10.6 min), geranial (rt: 10.9 min), citronellol (rt: 11.1 min), and nerol (rt: 11.3 min).

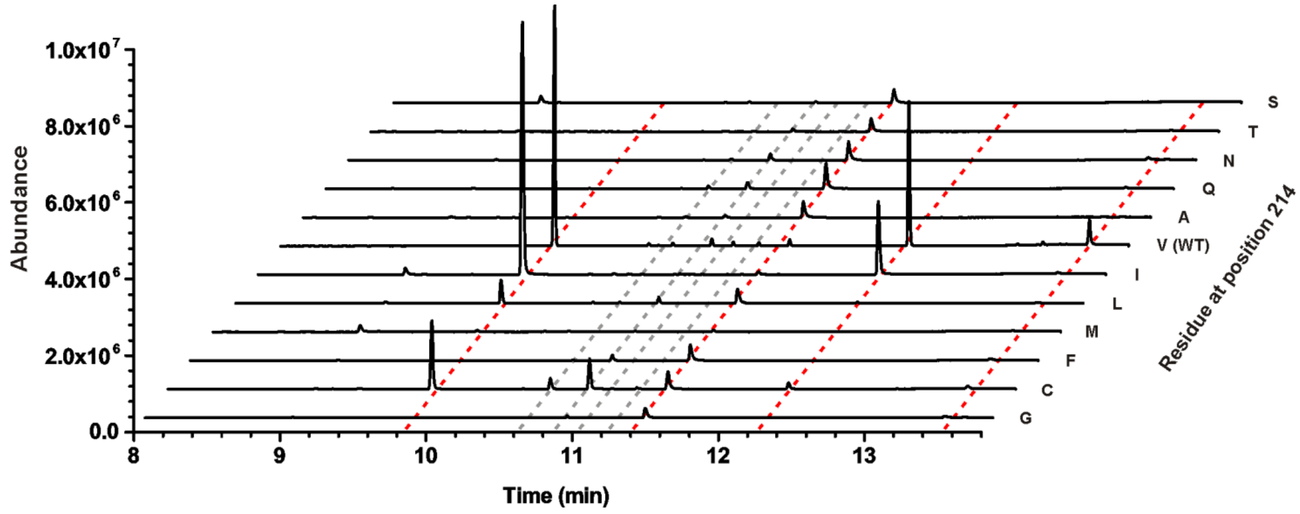

Figure S9: GC-MS analysis of bLinS-V214 variants. Representative total ion count chromatograms of bLinS-V214 variants when expressed in the *E. coli* monoterpenoid production platform[4].. Monoterpenoid peaks indicated with dashed red lines are: linalool (rt: 9.8 min), geraniol (rt: 11.4 min), *trans*-nerolidol (rt: 12.3 min), and farnesol (rt: 13.5 min). Compounds indicated with a dashed grey line are geraniol derivatives detected due to endogenous *E. coli* activity[4], and include neral (rt: 10.6 min), geranial (rt: 10.9 min), citronellol (rt: 11.1 min), and nerol (rt: 11.3 min).

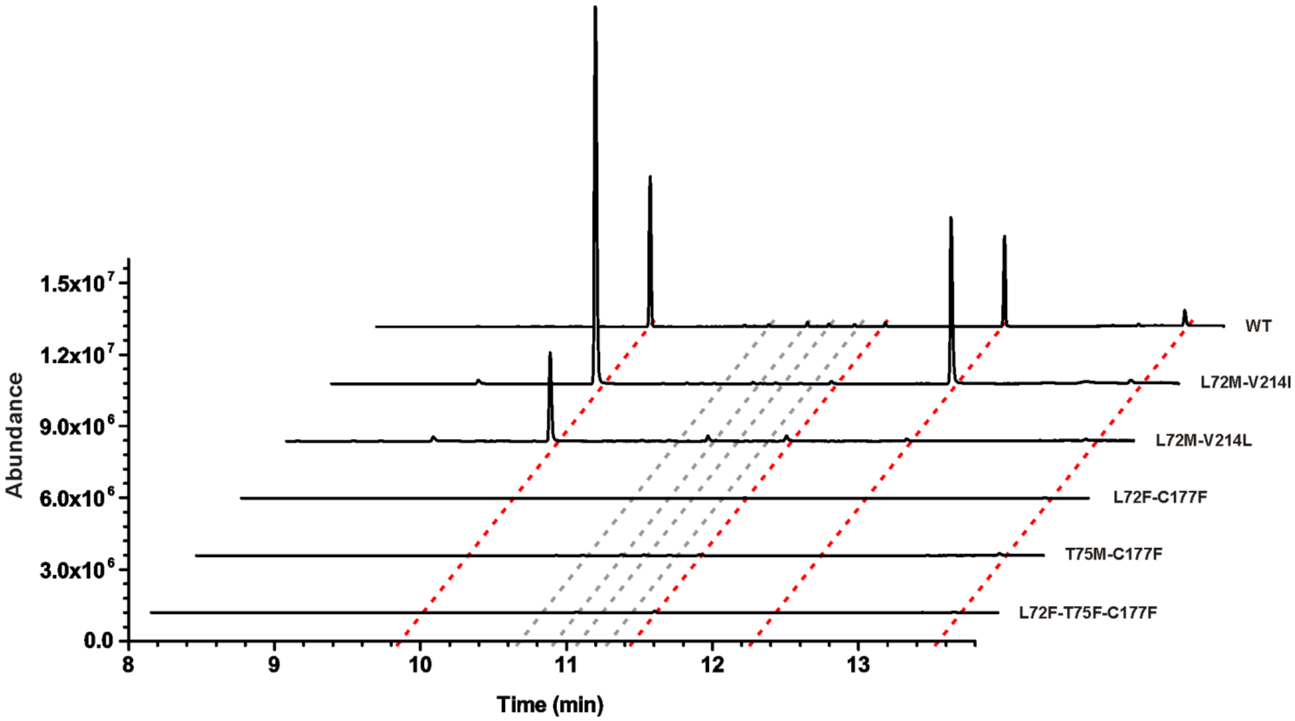

Figure S10: GC-MS analysis bLinS variants with multiple mutations. Total ion count chromatograms of wild-type bLinS, bLinS-L72M-V214I, bLinS-L72M-V214L, bLinS-L72F-C177F, bLinS-T75F-C177F, and bLinS-L72F-T75F-C177F when expressed in the *E. coli* monoterpenoid production platform[4]. Monoterpenoid peaks indicated with dashed red lines are: linalool (rt: 9.8 min), geraniol (rt: 11.4 min), *trans*-nerolidol (rt: 12.5 min), and farnesol (rt: 13.5 min). Compounds indicated with a dashed grey line are geraniol derivatives detected due to endogenous *E. coli* activity[4], and include neral (rt: 10.6 min), geranial (rt: 10.9 min), citronellol (rt: 11.1 min), and nerol (rt: 11.3 min).

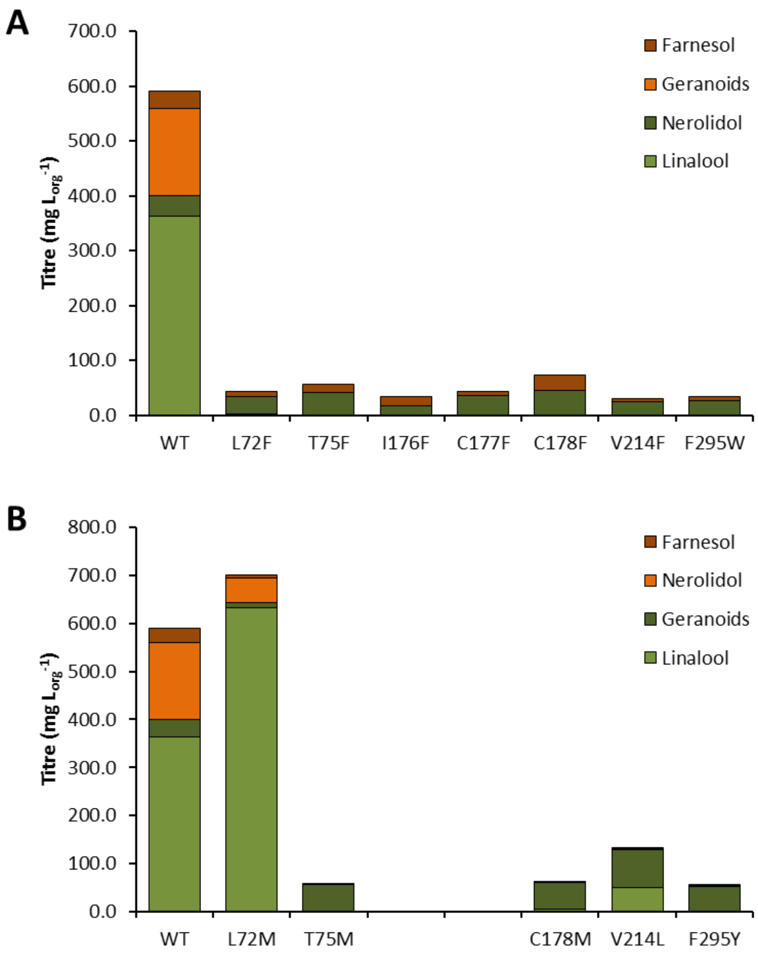

Figure S11: Comparison of product profiles wild-type bLinS (WT) and first round variants. A) Variants generated in the first round of mutagenesis. **B)** Variants generated in the second round of mutagenesis. Full product profiles, titres, and standard deviations are shown in Table S6. WT is wild-type bLinS.

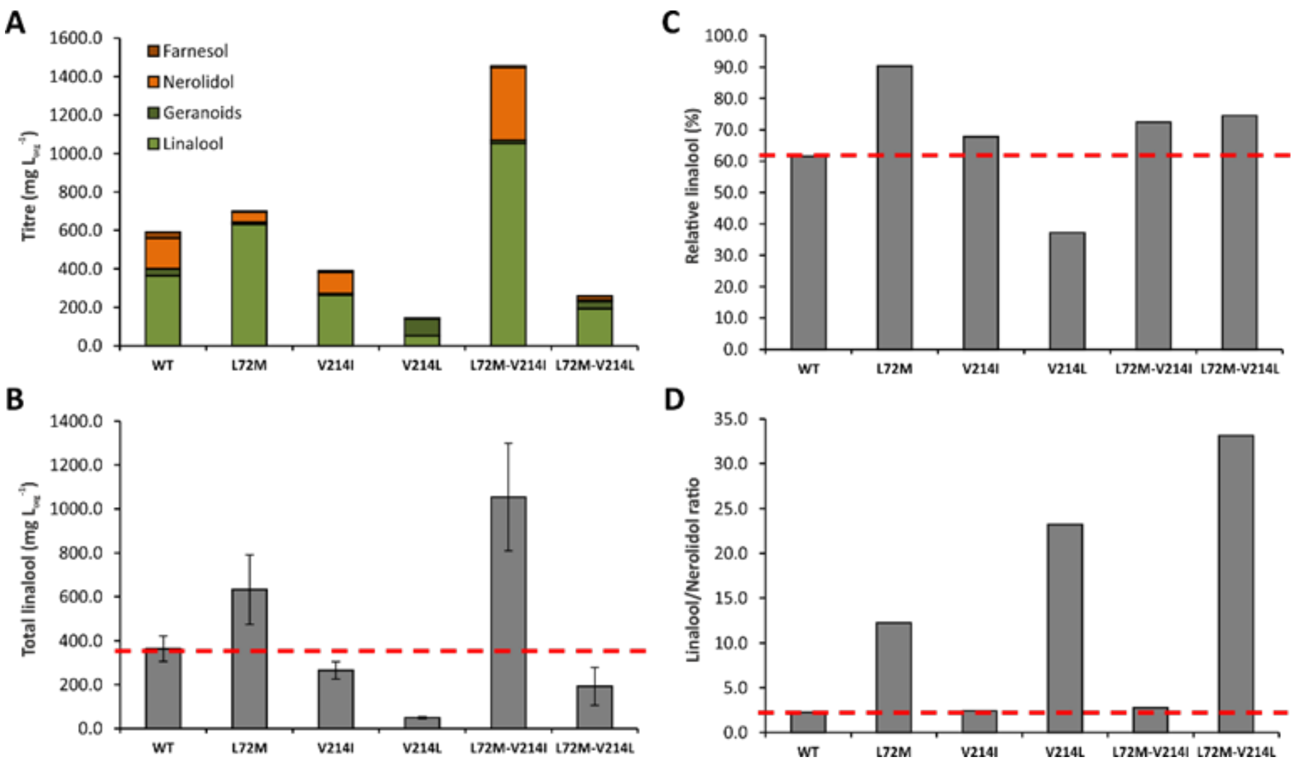

Figure S12: Properties of best performing bLinS variants when expressed in engineered *E. coli* for terpenoid production. A) Average product profiles and titres obtained. (B) Average linalool titres obtained. (C) Relative linalool content. (D) Linalool/nerolidol ratio. The dashed red line indicates which variants have favourable properties over wild-type bLinS. Full product profiles, titres, and standard deviations are shown in Table S6 of the Supplementary Information. WT is wild-type bLinS.

REFERENCES

[1] J. Alonso-Gutierrez, R. Chan, T. S. Batth, P. D. Adams, J. D. Keasling, C. J. Petzold, T. S. Lee, *Metab. Eng.* **2013**, *19*, 33-41.

[2] V. Karuppiah, K. E. Ranaghan, N. G. H. Leferink, L. O. Johannissen, M. Shanmugam, A. Ni Cheallaigh, N. Bennett, L. Kearsey, E. Takano, J. Gardiner, M. W. Van der Kamp, S. Hay, A. J. Mulholland, D. Leys, N. S. Scrutton, *ACS Catal.* **2017**, *7*, 6268-6282.

[3] A. O. Chatzivasileiou, V. Ward, S. M. Edgar, G. Stephanopoulos, *Proc Natl Acad Sci U S A* **2019**, *116*, 506-511.

[4] N. G. H. Leferink, A. J. Jervis, Z. Zebec, H. S. Toogood, S. Hay, E. Takano, N. S. Scrutton, *ChemistrySelect* **2016**, *1*, 1893-1896.

[5] C. Wang, S. H. Yoon, A. A. Shah, Y. R. Chung, J. Y. Kim, E. S. Choi, J. D. Keasling, S. W. Kim, *Biotechnol Bioeng* **2010**, *107*, 421-429.
